# Organic Nanoparticles in Progressing Cardiovascular Disease Treatment and Diagnosis

**DOI:** 10.3390/polym16101421

**Published:** 2024-05-16

**Authors:** Alexandru Scafa Udriște, Alexandra Cristina Burdușel, Adelina-Gabriela Niculescu, Marius Rădulescu, Paul Cătălin Balaure, Alexandru Mihai Grumezescu

**Affiliations:** 1 Department 4 Cardio-Thoracic Pathology, “Carol Davila” University of Medicine and Pharmacy, 050474 Bucharest, Romania; alexandru.scafa@umfcd.ro; 2 Department of Science and Engineering of Oxide Materials and Nanomaterials, National University of Science and Technology Politehnica Bucharest, 011061 Bucharest, Romania; alexandra.burdusel@upb.ro (A.C.B.); adelina.niculescu@upb.ro (A.-G.N.); agrumezescu@upb.ro (A.M.G.); 3 Research Institute of the University of Bucharest—ICUB, University of Bucharest, 050657 Bucharest, Romania; 4 Department of Inorganic Chemistry, Physical Chemistry and Electrochemistry, National University of Science and Technology Politehnica Bucharest, 1-7 Polizu St., 011061 Bucharest, Romania; marius.radulescu@upb.ro; 5 Department of Organic Chemistry, National University of Science and Technology Politehnica Bucharest, 1-7 Polizu St., 011061 Bucharest, Romania

**Keywords:** cardiovascular diseases, nanomedicine, organic nanoparticles, liposomes, micelles, dendrimers, polymeric nanoparticles

## Abstract

Cardiovascular diseases (CVDs), the world’s most prominent cause of mortality, continue to be challenging conditions for patients, physicians, and researchers alike. CVDs comprise a wide range of illnesses affecting the heart, blood vessels, and the blood that flows through and between them. Advances in nanomedicine, a discipline focused on improving patient outcomes through revolutionary treatments, imaging agents, and ex vivo diagnostics, have created enthusiasm for overcoming limitations in CVDs’ therapeutic and diagnostic landscapes. Nanomedicine can be involved in clinical purposes for CVD through the augmentation of cardiac or heart-related biomaterials, which can be functionally, mechanically, immunologically, and electrically improved by incorporating nanomaterials; vasculature applications, which involve systemically injected nanotherapeutics and imaging nanodiagnostics, nano-enabled biomaterials, or tissue-nanoengineered solutions; and enhancement of sensitivity and/or specificity of ex vivo diagnostic devices for patient samples. Therefore, this review discusses the latest studies based on applying organic nanoparticles in cardiovascular illness, including drug-conjugated polymers, lipid nanoparticles, and micelles. Following the revised information, it can be concluded that organic nanoparticles may be the most appropriate type of treatment for cardiovascular diseases due to their biocompatibility and capacity to integrate various drugs.

## 1. Introduction

Globally, cardiovascular diseases (CVDs) are becoming more common, being the primary cause of premature mortality and disability in humans [1,2]. In addition to significantly raising health care expenses, CVDs place a significant socioeconomic burden on the general populace [3,4,5]. This causes peripheral vascular disease, venous thromboembolism, coronary artery disease, and cerebrovascular disease, which can all lead to myocardial infarction, cardiac arrhythmias, or stroke [5,6].

The aetiological risk factors that contribute to the development of CVDs are well acknowledged and comprise obesity, smoking, diabetes, hypertension, hyperlipidemia, and physical inactivity. Combined, they account for almost 90% of the risks of CVD in all epidemiological studies. Notwithstanding the high death rate associated with CVDs, the global epidemic of these diseases can be considerably curbed by identifying and carefully preventing the underlying risk factors [7].

**Figure 1:**
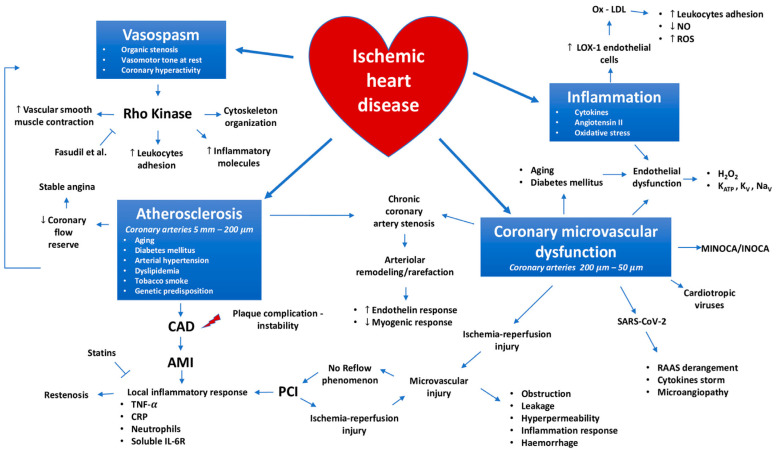
Diagrammatic illustration of the pathophysiological processes behind ischemic heart disease. Abbreviations: CAD: coronary artery disease; AMI: acute myocardial infarction; PCI: percutaneous coronary intervention; TNFα: tumor necrosis factor alpha; CRP: C-reactive protein; IL-6R: interleukin-6 receptor; SARS-CoV-2: severe acute respiratory syndrome coronavirus 2; RAAS: renin–angiotensin–aldosterone system; MINOCA: myocardial infarction with non-obstructive coronary arteries; INOCA: ischemia with non-obstructive coronary arteries; H_2_O_2_: hydrogen peroxide; K_ATP_: ATP-sensitive potassium channel; K_v_: voltage-gated potassium channel; Na_v_: voltage-gated sodium channel; LOX-1: oxidized low-density lipoprotein receptor 1; Ox-LDL: oxidized low-density lipoprotein; ROS: reactive oxygen species; NO: nitric oxide; Symbols: ↑: increase; ↓: decrease. Reprinted from an open-access source [8].

The traditional pathophysiological theory of ischemic heart disease (IHD) (or coronary artery disease (CAD)) states that myocardial ischemia is caused by an obstructive atherosclerotic plaque that restricts blood flow via the coronary artery. These issues can be linked to several processes, as depicted in Figure 1. An imbalance in the communication between myocardial energy status and coronary blood flow is the determining factor in IHD, with atherosclerosis, coronary microvascular dysfunction, vasospasm, and inflammation being the main factors that contribute to the intricate and complicated pathophysiology of ischemic heart disease [8].

Primary regimens for preventing and treating CVDs include lipid-lowering drugs, antihypertensives, antiplatelet, and anticoagulant treatments, in addition to making positive lifestyle changes [5,9,10,11]. Despite the existence of several therapeutic options, there is still room for improvement in CVD management toward providing better results for affected patients. Specifically, the administration of conventional pharmaceutical treatments for CVD has several disadvantages [12]. Because in vivo active agents have short half-lives and nonspecific distributions, systemic delivery of these agents frequently necessitates high concentrations. Adverse toxic side effects, unsustainable drug levels, and the development of drug resistance can result from these levels [13].

In this respect, new treatment perspectives with great promise for better outcomes than traditional therapeutic strategies have been envisaged with the aid of nanotechnology [14,15]. Materials with overall dimensions in the nanoscale, referred to as nanoparticles (NPs), have become significant players in contemporary medicine in recent years, with uses ranging from carriers of genes into particular cells to contrast chemicals in medical imaging [16,17].

The three main types of NPs of interest for biomedical applications are organic, inorganic, and hybrid nanostructures. Nano-sized vesicles are highly relevant in managing CVDs, as they can improve myocardial recovery in ischemic heart diseases and function as sustained-release delivery systems for therapeutic agents [18,19]. Proteins, carbohydrates, lipids, and other organic compounds are used to create organic nanoparticles frequently used in cardiac therapy [19].

**Figure 2:**
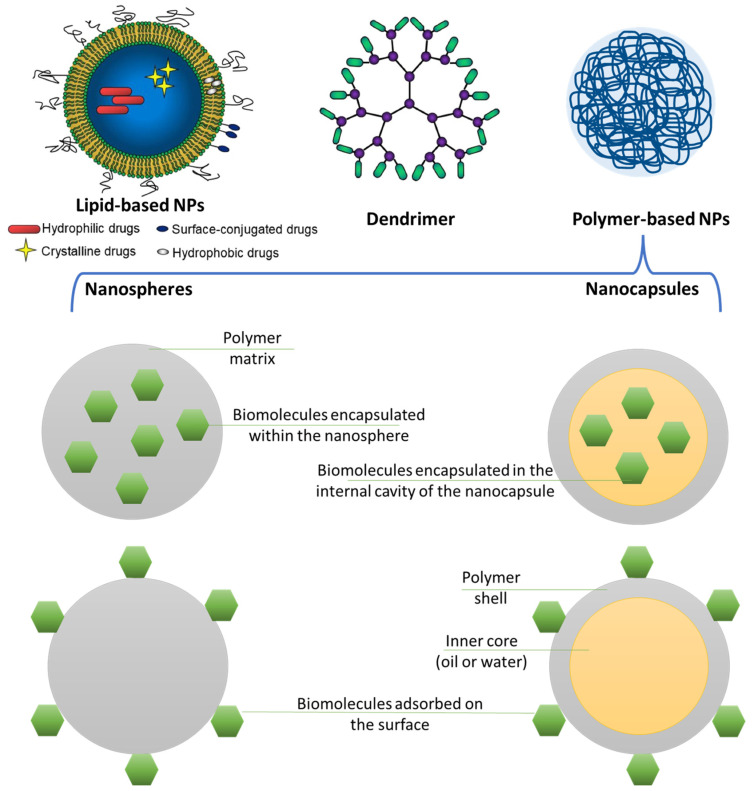
Types of organic nanoparticles (NPs) that are frequently employed in biomedical applications. Adapted from open-access sources [19,27].

With the help of NPs, systemic drug inefficiency can be improved, bleeding and other problems can be greatly reduced, and tissue plasminogen activator (t-PA) [20] and other thrombolytic drugs can be delivered with precision [21,22,23]. As it is crucial to restrict exposure to off-target sites for all CVD therapies, modularly constructed organic-based nanoplatforms can be employed to decrease side effects and enhance therapeutic efficacy [24]. By focusing on specific organs, cells, and disease pathways, nanotechnology improves the accuracy of diagnoses and guides tailored treatment. One of the most important gabs in the present treatment of CVD may be represented by the ability to identify and target certain diseased sites, such as inflammation, thrombosis, or proliferation inside the heart or blood arteries, without damaging healthy tissues. Nanomedicine can provide important tools with various exciting possibilities for treating pathologies particular to a place without causing systemic adverse effects. They also make it possible for platform nanotechnologies to provide multifunctional therapeutic and/or diagnostic functionality as well as “all-in-one” theranostics [25]. Thus, numerous studies have focused on developing and evaluating the performance of various biocompatible nanostructures for optimizing CVD therapeutic outcomes. Researchers worldwide have explored the use of different lipid and polymeric-based nanomaterials to find better treatment alternatives [26] (Figure 2). Liposomes, natural and synthetic polymeric nanoparticles, dendrimers, and lipid and polymer-based micelles have been evaluated in literature studies with encouraging results.

In this context, the following sections describe the applications of organic nanoparticles in CVDs, emphasizing the advances made in this interdisciplinary research area and setting an updated background for further studies. In more detail, recent English-language publications focused on lipid-, micelle, and polymer-based nanoformulations relevant to CVD management have been reviewed in this paper.

## 2. Lipid Nanoparticles

### 2.1. Types of Lipid-Based Nanoformulations and Their Relevant Features for CVDs

Frequently employed lipid-based NP formulations in nanomedicine include liposomes, solid lipid nanoparticles, nanostructured lipid carriers, lipid–drug conjugates, and nanoemulsions [28]. Physiological lipid analogs with surfactants acting as stabilizers comprise most of these formulations [18]. Lipids and other artificially amphiphilic molecules can be tuned to self-assemble in an aqueous solution (as a function of temperature, pH, ionic force, etc.), leading to the formation of micelles (i.e., monolayers containing a hydrophilic head and hydrophobic tail) [29]. Compared to liposomes, the enclosed space in a micelle is more constrained. Liposomes are the first in nanomedicine to receive FDA approval and are the subject of extensive research. Phospholipids, which comprise most liposomes, form bilayers with an aqueous phase inside, giving the liposomes superior biocompatibility [30,31,32].

Lipid nanoparticles are a promising option for administering drugs to the myocardium. They can include hydrophilic and lipophilic materials, and their morphology is comparable to cell membranes [33]. They have effectively displayed the capacity to introduce a variety of biomaterials, including peptides, proteins, nucleic acids, imaging agents, and low molecular weight drugs, into the intended tissue [34]. Clinical siRNA administration using lipid nanoparticles (LNPs) is a potentially effective drug delivery method. Recently, there has been a lot of interest in modified mRNA (modRNA) as a potential therapeutic molecule for heart regeneration. To be functional, mRNA must first pass through the target cell, exit the endosomal compartment, enter the diseased heart, and be translated into a working protein in the cytosol. However, LNPs may not be able to successfully transport mRNA, which is substantially larger than siRNA, to the ischemic myocardium [18].

It was found that micelles can enter the entire infarct area, an ability that renders them promising delivery systems for drugs that control infarct healing during the chronic stage of myocardial infarction (MI) and cardioprotective drugs required for the acute stage. However, liposomes are considered better candidates for the delivery of pro-angiogenic drugs since they provide slower and more constrained extravasation from the vasculature. Moreover, the non-invasive magnetic resonance imaging (MRI) visualization of liposomes and micelles may provide a flexible strategy for the creation of efficient cardioprotective treatment approaches [19,35,36].

Getting a pharmaceutical to the injured area is the common aim of liposome therapy and diagnostic applications. Liposomes are spherical, self-closing structures composed of one or more concentric lipid bilayers with an aqueous phase between and within the lipid bilayers [37]. Their capacity to entrap various water-soluble substances in the inner aqueous phase and lipophilic agents in between liposomal bilayers has rendered them valuable for drug delivery applications as well as for the transportation of diagnostic agents in all imaging modalities, including MRI, CT, gamma-scintigraphy, and sonography [38,39,40]. Liposomes are small vesicles with a diameter of 20 nm to 2.5 μm. They could be fabricated from one or more non-concentric or concentric membranes. Acute parameters may influence drug encapsulation efficiency and half-life in circulation, including vesicle size and number of bilayers. Liposomes can be categorized as unilamellar vesicles, multilamellar vesicles (MLV, >500 nm), or multivesicular vesicles (MVV, >1000 nm) based on the size and quantity of bilayers [41]. The first instance of an effective medication delivery method utilizing nanoparticles is liposome-encapsulated doxorubicin (Doxil), which is used to treat AIDS-related Kaposi’s sarcoma, multiple myeloma, and ovarian cancer [42,43]. Since then, noticeable progress has been made, and liposomes started being explored for numerous other biomedical applications, including alternative therapeutics for CVDs.

Nanoparticle production has also made considerable use of block copolymers. The size and form of the nanoparticles can be altered by varying the lengths of block copolymers’ hydrophilic and hydrophobic components. Moreover, selecting a certain block co-polymer can regulate the release rate and degradation [44].

Because liposomes are highly biocompatible, biodegradable, and immunogenicity-free, they are now thought to be the most widely employed nanocarriers for a variety of hydrophobic and hydrophilic compounds that have the potential to be active. Moreover, liposomes have demonstrated improved drug solubility, regulated dispersion, and surface modification ability for focused, extended, and sustained release. Liposomes can be thought of as having developed from conventional, long-circulating, immune-targeting liposomes to stimuli-responsive, actively targeted liposomes based on their composition [45].

Liposomal modification with polyethylene glycol (PEG) expands their field of application by improving circulation time and allowing antibodies or other targeting moieties to adhere to their surface to target particular afflicted areas [46]. Functionalization agents, such as cells and noncellular components (e.g., endothelial cells, subendothelial structures, blood components, monoclonal antibodies, and antibody fragments) act as targeting entities for the diagnosis and treatment of the most significant cardiac pathologies, including myocardial infarction, coronary thrombosis, and atherosclerosis, especially given that such modified liposomes and immunoliposomes can be considered for intravascular drug delivery [47,48,49,50].

Despite the lack of commercially available liposomal drug formulations for the treatment of CVD, liposomes have made significant steps in improving the effectiveness of cardiovascular drug delivery in the context of in vivo animal studies. For instance, liposomes changed lipid metabolism in small and large animal models by delivering small interfering ribonucleic acid (siRNA) to suppress the expression of proprotein convertase subtilisin–kexin type 9 (PCSK9) and apolipoprotein B. This resulted in a decrease in low-density lipoprotein levels [50,51,52]. Interesting possibilities have also been envisaged for using lipid nanoparticles for messenger RNA delivery (mRNA) with application in various CVDs [53]. Specifically, several recent studies have explored the synergy of mRNA and lipidic nanocarriers for improving cardiac function and regeneration [54], decreasing low-density lipoprotein cholesterol levels [55], and treating endotheliopathy associated with vascular senescence [56,57].

### 2.2. Liposomes—Drug/Gene Delivery Systems

Liposomes are an excellent option for drug encapsulation that can be administered with less pain during injection because of their special qualities, which include biodegradability, low toxicity, high carrying capacity, and ease of preparation [32,58]. For instance, istaroxime, a potentially effective and safe treatment for acute and chronic heart failure, is delivered by vesicles formulated with phospholipids and PEG-HS, an excipient selected to modulate the bilayer properties [47].

Additionally, cardiovascular intoxication can be treated with liposomes because of their capacity to sequester substances from the bloodstream and release the encapsulated drug. The development of long-circulating liposomes with a transmembrane pH gradient has allowed for the demonstration of the drug’s in vivo scavenging properties through pharmacokinetic analysis as a scavenging nanocarrier for diltiazem intoxication [47]. Many studies have proven that modifying the inflammatory response by alternate macrophage polarization can provide protection against acute myocardial infarction (MI)-related consequences. Prior to MI, oral azithromycin (AZM) has been demonstrated to decrease inflammation and its detrimental effects on the myocardium. To improve the therapeutic potency and prevent off-target effects, Al-Darraji et al. [59] examined the immunomodulatory function of a liposomal AZM formulation (L-AZM) in a model that is relevant to clinical practice. Following MI, L-AZM (40 or 10 mg/kg, IV) was given and compared to free AZM (F-AZM). In mice, L-AZM lowered heart toxicity and related mortality by 50%. With L-AZM formulation, it was observed a notable shift favoring reparatory/anti-inflammatory macrophages. The usage of L-AZM has shown an improved reduction in the infiltration of inflammatory monocytes and cardiac inflammatory neutrophils [59].

Liposomes can be used with polymer-coated stents or as a stand-alone drug delivery mechanism. Stents coated with polymeric material containing dispersed or encapsulated drugs have been developed as a method to prevent stent-related complications in a non-invasive manner [60]. The possibility of coating polymer-covered stents with liposomes encapsulating heparin to increase the hemocompatibility of polymer-covered stents was explored. The biological functionality of encapsulated heparin was maintained, and the release duration was contingent upon the liposome preparation technique and lipid composition [61]. The same approach was used to create the most recent drug-eluting stents to demonstrate the potential for gene delivery.

Brito et al. created a gene-eluting stent with liposome/DNA complexes immobilized on the stainless steel surface to lessen coronary restenosis. Studies conducted in vivo using a rabbit model of iliac artery restenosis revealed that green fluorescent protein expression in arterial tissues appeared 24 h after implantation [62].

According to Allijn et al.’s study [63], encapsulating berberine into lengthy circulating liposomes may increase the drug’s therapeutic availability and efficacy by shielding the heart from MI in vivo. By injecting ethanol, berberine-loaded liposomes were created and analyzed. Comparing berberine-loaded liposomes to control liposomes and free berberine, the cardiac ejection fraction at day 28 after MI was considerably conserved by 64% in vivo. The study concluded that liposomal encapsulation maintains ejection fraction during MI and improves berberine’s solubility in the buffer. This demonstrates how berberine-loaded liposome administration greatly increases their therapeutic availability and suggests that they may be used to treat unfavorable remodeling following myocardial infarction [63].

It is possible to improve gene delivery to targeted cells by combining increased intracellular penetration with specificity and specific anti-myosin antibodies. Double-targeted delivery systems were first used in vitro when liposome plasmid DNA complexes modified with cell-penetrating transactivating transcriptional activator peptide (TATp) and/or monoclonal anti-myosin antibody were tested with normoxic and hypoxic H9C2 cardiomyocytes. The presence of TATp and the additional modification with antibodies improved transfection under both circumstances. An enhanced transfection of cardiomyocytes in the ischemic zone and an increased accumulation of immunoliposomes modified with TATp were clearly observed in the in vivo rat study with experimental MI [64].

In the Langendorff isolated rat heart model, Verma et al. [65] showed that optimized and targeted liposomes loaded with ATP can produce cardioprotective effects ex vivo. After 30 min of reperfusion, ATP-liposomes (ATP-L), injected one minute before the onset of global ischemia significantly protected the ischemic myocardium. When using ATP-L (61%) as opposed to the Krebs–Henseleit (KH) buffer, the left ventricular end-diastolic pressure was significantly lower after reperfusion. The left ventricular developed pressure considerably recovered to above 80% of the baseline when myosin-specific monoclonal antibodies were also attached to the liposomes, as opposed to 25% in the KH buffer group. The quantity of the antibody bound to the liposomal surface also determined this protective effect. For an in vivo investigation, rabbits with induced MI were given ATP-L. Intracoronary liposome infusion was used, along with occlusion for 30 min and reperfusion for 3 h. Approximately 30% of the area at risk has experienced irreversible damage in the ATP-L-treated animals, compared to roughly 60% in the empty liposome-treated group and roughly 70% in the KH buffer-treated group [65,66].

To promote inflammation resolution and induce infarct repair, Harel-Adar et al. [67] studied a novel method for modifying cardiac macrophages to a reparative state at a predefined time following myocardial infarction (MI). Phosphatidylserine (PS)-presenting liposomes were injected intravenously as part of the effort to simulate the anti-inflammatory properties of apoptotic cells. Both in vitro and in vivo, macrophages that had taken up PS-liposomes secreted high amounts of anti-inflammatory cytokines, such as transforming growth factor β (TGFβ) and interleukin 10 (IL-10), and they also upregulated the expression of the mannose receptor, CD206, while downregulating proinflammatory markers, such as surface marker CD86 and tumor necrosis factor α (TNFα). Targeting PS-presenting liposomes to infarct macrophages following injection via the femoral vein was shown in a rat model of acute MI by using MRI [67]. Injectable scavenging nanocarriers have been proposed by Bertrand et al. [68] as detoxifying agents in the absence of specific antidotes for pharmacological overdoses. They limit the drug’s distribution in tissues by trapping it there. Despite their relatively low absorption capacity, parenteral lipid emulsions are the only systems utilized in clinics for that purpose. Long-circulating liposomes with a transmembrane pH gradient were analyzed in this study to treat diltiazem intoxication. The drug was sequestered in the bloodstream, and its pharmacological effect was limited by the vesicles’ special ion-trapping abilities toward ionizable compounds. Following formulation optimization in vitro, the drug’s pharmacokinetics were examined to illustrate the liposomes’ capacity for in vivo scavenging. Limited tissue distribution was indicated by the animals treated with liposomes, as evidenced by the decreased volume of distribution and increased area under the plasma concentration versus time curve. The main active metabolite of the drug, deacetyl-diltiazem, was affected by the vesicles in a comparable but more noticeable manner. This drug and metabolite uptake in vivo changed the overall pharmacological result. For one hour, the liposomes prevented the hypotensive decline and preserved a higher average blood pressure in rats given an intravenous diltiazem bolus. When the rats were given larger doses of the drug through perfusion, the detoxifying effect of the liposomes was even more pronounced. As a result, the mentioned study validated the potential of liposomes as effective detoxifying nanocarriers by demonstrating their ability to alter the pharmacokinetics and pharmacodynamics of diltiazem and its metabolite [68].

Differently, Chen and colleagues [61] synthesized biomimetic nanoparticles for the targeted and all-encompassing anti-inflammatory treatment of MI. Biomimetic liposomes (Neu-LPs) were created by fusing neutrophil membranes with conventional liposomes. These liposomes retained the surface antigens of the source cells, which allowed them to function as perfect semblances of biological molecules that target neutrophils. Neu-LPs targeted infarcted hearts and neutralized proinflammatory cytokines, suppressing intense inflammation and regulating the immune microenvironment due to their abundant chemokine and cytokine membrane receptors. Consequently, in a mouse model of myocardial ischemia-reperfusion, Neu-LPs demonstrated a strong therapeutic benefit by protecting the heart and stimulating angiogenesis. As a result, Neu-LPs have a great potential for clinical translation and may be created as an anti-inflammatory drug to eliminate inflammatory cytokines with a broad spectrum during MI and other disorders involving neutrophils [61].

Targeting nanocarriers with monoclonal antibodies has already been shown to work. Despite numerous encouraging reports, its use as an antibody carrier system has not yet been authorized for clinical use. Liposomes modified with particular monoclonal antibodies against different targets are a major feature of the tumor vasculature and cardiovascular system. Creating immunoliposomes involves conjugating antibodies to either the distal end of the liposomal polyethylene glycol or its surface. Myosin antibody fragment localization and specificity have previously been shown in experimental myocardial infarction, where the antibody activity was maintained after covalent coupling [69].

Various methods, such as coating liposomes with biocompatible, inert polymers like poly lactic-co-glycolic acid (PLGA) or PEG, have been tried in order to achieve an in vivo sustained release profile for the drugs. The coating keeps the liposomes safe from being cleared after an opsonin-based reorganization. In addition, much research is currently being conducted on long-circulating liposomes to determine their potential uses for in vivo and biomedical studies. According to studies, a 4–10% PEG growth on the liposome surface lengthens its in vivo circulation time from 200 to 1000 min. Proteins, peptides, and other molecules can be chemically conjugated to the liposomes’ outer surface to create modified liposomes with the required controlled properties [70]. Liposomes can also be made to be pH-sensitive by utilizing pH-sensitive components, allowing for a pH-dependent release of drugs from the vesicles. The reason for this is that the endosomes’ pH is lower. In this setting, liposomes tend to fuse with the endovascular membrane, causing the contents to be released into the cytoplasm. Diagnostic liposomes are primarily used to target MI and other CVDs. These liposomes aid in repairing the cardiocyte’s damage and sealing the hypoxia-induced plasma membrane. It has also been discussed that their relative significance for the size, targetability, and sustained circulation time efficiency represents a promising therapeutic strategy [58].

A study led by Dorostkar et al. [71] examined the physicochemical features of liposomal doxorubicin, which was designed and produced using the thin-film approach with desirable qualities. Next, in animal models, the effects of quercetin, empty liposomes, free doxorubicin, liposomal doxorubicin, and the carrier were examined. Measurements of cardiac enzymes, oxidative stress and antioxidant indicators, protein expression, and histological investigations were carried out to assess the therapies. H9c2 cells were also used for cellular uptake and cytotoxicity assays. When rats were given liposomal doxorubicin and free quercetin together, their left ventricles’ activity of the enzymes glutathione peroxidase, catalase, and superoxide dismutase increased, and their levels of weight loss, creatine kinase (CK-MB), lactate dehydrogenase (LDH), and malondialdehyde (MDA) decreased [71].

Mukhamadiyarov et al. [39] studied the impact of ischemia/reperfusion injury on the myocardial accumulation of two populations of liposomes (diameters of approximately 70 and 110 nm). It is important to note that the size of the liposomes affects their cardioprotective and vasodilatory effects. Specifically, the population of liposomes with an average diameter of about 70 nm shows a more pronounced protective impact than the population with an average diameter of about 110 nm. The varying effects of liposomes based on size exhibit dosage dependence since smaller particles have the potential to penetrate capillaries more readily and concentrate in the ischemic myocardium [39].

### 2.3. Liposomes—Imaging Tools

Atherosclerosis is characterized by complex events involving multiple molecular factors, chemokines, and cell types. Since relatively small lesions can cause coronary events, such as myocardial infarction and stroke, early and accurate diagnosis is essential to start proper treatment and avoid acute complications. The current gold standard for diagnosing atherosclerosis is X-ray coronary arteriography; however, this invasive procedure frequently fails to identify susceptible atheromas because of negative remodeling of the vessel and may increase the risk of bleeding at the catheter insertion site. While more technological advancements are required before non-invasive imaging technologies like conventional ultrasound, CT, and MR can be widely used, they may provide lower-risk alternatives for characterizing atherosclerotic plaque. Using contrast agents, or tracers, has been one of the advances in MR angiography as it can decrease scan time, reduce artifacts, and improve image clarity [72,73]. Nanoparticles can decrease the toxicity of tracers and carry surface-targeting ligands to improve residence time and target cell/tissue delivery specificity, much like they can increase the efficiency of drug and gene delivery. Because liposomes have the unique capacity to increase residence time and stabilize contrast agents in the blood pool, they are especially interesting for this application [74].

A therapeutically useful method for imaging the total inflammatory cell burden in plaque has been developed by Woodisde et al. [75]. They described a targeted contrast agent (THI0567-targeted liposomal-Gd) that binds to the integrin α4β1 (very late antigen-4, or VLA-4) with high affinity and selectivity. This integrin is important for drawing inflammatory cells to atherosclerotic plaques. Because of the strong T1 relaxivity (~2 × 10^5^ mM^−1^s^−1^ on a particle basis) of this liposomal contrast agent, liposomes can be seen at a clinically meaningful MR field strength. Using a 1 Tesla small animal MRI scanner, they were able to see atherosclerotic plaques in several aortic areas in atherosclerosis-prone ApoE^−/−^ mice. The accumulation of monocytes and macrophages in the subendothelial layer of atherosclerotic plaques in vivo was correlated with these enhanced signals; non-targeted liposomal nanoparticles did not show a similar degree of signal augmentation. To noninvasively identify people at risk of an acute ischemia event, a method that targets inflammatory cells and has the specificity and sensitivity to quantify the inflammatory burden of a plaque may be employed [75].

To enhance MR angiography, Ayyagari et al. [76] enclosed gadodiamide in liposomes coated with polyethylene glycol. When compared to untargeted liposomal gadolinium, the study showed that liposomes lengthened the residence period in blood vessels with higher contrast. It has been suggested that inflammatory cells, like macrophages and monocytes, are good targets for imaging agents to increase residence time and homing accuracy because they actively contribute to developing atherosclerotic plaque and can absorb nanoparticles [76].

A liposomal nanoparticle containing surface-conjugated gadolinium (SC-Gd liposomes), a novel long-circulating blood-pool contrast agent, was created by Howles et al. and tested for application in mouse neurovascular magnetic resonance angiography. Twelve mice were scanned, and the scan parameters were tuned for SC-Gd contrast and time-of-flight. SC-Gd liposomes (0.08 mmol/kg) allowed for better small-vessel contrast-to-noise ratio, a bigger field of view, a shorter scan time, and the imaging of venous structures compared to time-of-flight contrast. As an alternative, high-resolution magnetic resonance angiography (0.27 nL) with a 32% higher contrast-to-noise ratio (*p* < 0.001) and a 75% shorter scan time (12 min) may be obtained using SC-Gd liposomes [77].

In another study, Maiseyeu et al. [78] designed liposomal gadolinium enriched with exteriorized phosphatidylserine residues, which are known to promote macrophage recognition and apoptosis improved the MR imaging signal of plaques in ApoE^−/−^ mice. Therefore, creating liposomes containing tracers specifically designed to draw iok7 macrophages to atherosclerotic lesions could make imaging easier and improve MR scanning resolution. Targeted liposomal imaging agents can also be advantageous for CT imaging [78].

For clarity, Table 1 offers an at-glance view of the most important liposome-based treatments for cardiovascular diseases.

## 3. Micelles

### 3.1. Types of Micelle-Based Nanoformulations and Their Relevant Features for CVDs

Micelles are nanoscale colloidal particles with a hydrophilic shell and a hydrophobic core designed to be effective carriers for poorly soluble drugs. Micelle-based NPs can be shaped into different morphologies; the most common structure is spherical. When lipids or amphiphilic molecules are dissolved in water, micellar structures can self-assemble. The shell is a barrier between the inner core and the outside world, while the inner core is where insoluble drugs are loaded. Micelles have been employed extensively due to their good biocompatibility, long retention period, easy production, and high drug encapsulation effectiveness. Micelles are more compact in spatial structure, smaller in size, and have a lower loading capacity than liposomes [80].

Because micelles are more oriented toward the lesions, the ischemic myocardium is more permeable to them. Micelles are generally involved in delivering drugs, but they can also target particular plaque components [81]. A modular multifunctional micelle containing antithrombin was created by Peters et al. [82]. It first targets atherosclerotic plaques and then binds to clotted plasma proteins. It was observed that the targeted micelles delivery system decreased the risk of plaque rupture and raised antithrombin activity in diseased vessels [82].

### 3.2. Micelles—Drug/Gene Delivery Systems

Surface-modified polymeric micelles mainly comprise amphiphilic macromolecules and have been widely used in drug delivery and theranostics [83]. Polymeric micelles have been prepared using a variety of polysaccharides. The capacity of the encapsulated therapeutic drugs to reach the intended location depends critically on the stability of the micelles in the circulatory system. Nevertheless, upon intravenous injection, micelles are typically not stable in the presence of proteins and ions. The stability of micelles is significantly influenced by the polymer chain structure on their surface [84].

Polyethylene glycol (PEG)-based micelles containing gadolinium diethylenetriamine pentaacetic acid (Gd-DTPA) amphiphile were designed by Belivert et al. [85] as magnetic resonance contrast agents. The micelles were functionalized with tyrosine residues, an aromatic, lipophilic amino acid, in a lipoprotein-inspired method to effectively reach the lipid-rich regions of atherosclerotic plaque. These micelles were used as an atherosclerosis model on apolipoprotein E^−/−^ (ApoE^−/−^) mice. The abdominal aortic wall was significantly improved by PEG micelles modified with 15% tyrosine residues at 6 and 24 h postinjection (pi) compared to unmodified micelles. A relation between the distribution of the functionalized contrast agent in plaque and lipid-rich regions was seen using fluorescence microscopy on histological sections of the abdominal aorta. It was shown that lipid-rich regions in the ApoE^−/−^ mice’s atherosclerotic plaque can be identified by MRI employing Gd-DTPA micelles using a straightforward method [85].

A PEG-based lipid micelle system co-encapsulated with a fluorophore as an imaging agent was shown by Cormode et al. to be able to deliver an anticoagulant drug at the same targeting site [86]. Additional research revealed the effectiveness of PEG-based lipid micelles encapsulated in gadolinium (Gd) complex and surface-modified scavenger receptor-based antibodies to specifically accumulate in atherosclerotic arterial sites. Gd-encapsulated micelles functionalized with anti-CD36 antibodies could identify macrophages in human atherosclerotic aortic tissues taken during autopsy at atherosclerotic aortic sites [87]. Unfortunately, one of the effects of PEG coating may be the CARPA effect. Acute hypersensitivity responses (HSRs) can occur in as many as 45% of patients after intravenous injection of some liposomal medications, diagnostic tools, micelles, and other lipid-based nanoparticles [88]. These reactions can have hemodynamic, respiratory, and cutaneous symptoms. Recent studies have produced strong evidence that complement (C) activation by the lipid particle is the cause of many of these HSRs, also known as “infusion” or “idiosyncratic” reactions. As such, the syndrome was named “nonallergic drug hypersensitivity” with a new subclass called “C activation-related pseudoallergy” (CARPA) [89]. Several potential micelle-based treatments for dysfunctional endothelial, which are a major component of thrombotic or atherosclerotic tissues, were investigated by Nakashiro et al. [90]. Their findings demonstrated the great promise of micellar site-specific delivery and its applicability in managing CVDs [90].

Resident vascular smooth muscle cells (VSMCs) in the blood arteries may encounter phenotypic flipping from the quiescent, contractile phenotype to the migratory, proliferative, synthetic phenotype in atherosclerosis, becoming highly plastic. Furthermore, it has been discovered that VSMCs transdifferentiate into osteochondrogenic and macrophage-like cells, making up to 70% of the cells in atherosclerotic plaques in recent VSMC lineage-tracing mice models of atherosclerosis. It has been proven that microRNA-145 (miR-145) controls phenotypic switching in VSMCs; Chin et.al. hypothesized that delivering miR-145 to VSMCs by nanoparticle delivery could slow the progression of atherosclerosis by preventing the growth of plaque-propagating VSMC-derived cell types. Thus, they designed miR-145 micelles to target the C-C chemokine receptor-2 (CCR2), which is highly expressed in artificial VSMCs. In vitro, incubation of miR-145 micelles with human aortic VSMCs resulted in >90% of the micelles escaping the lysosomal route within 4 h and releasing the miR cargo under the influence of endogenous reducing agent glutathione present in the cytosol. In vitro, miR-145 micelles restored the atheroprotective contractile markers, α-SMA, calponin, and myocardin, in synthetic VSMCs [91].

Wennink et al. [92] studied photodynamic therapy’s ability to specifically eliminate macrophages to potentially lower atherosclerotic plaques. As a photosensitizer, temoporfin or m-tetra(hydroxyphenyl)chlorin (mTHPC) may be used to treat cancer and atherosclerotic lesions. This study used benzyl-PCL-b-methoxy PEG to create polymeric micelles that were then loaded with mTHPC using the film hydration method. The authors reported that after 30 min, the mTHPC was delivered in blood plasma from the micelles and that the accumulation of mTHPC in mice’s atherosclerotic lesions led to its release from the micelles and binding to lipoproteins [92]. Subsequent studies could enhance the stability and accumulation of Ben-PCL-mPEG micelles loaded with THPC in macrophages residing in atherosclerotic lesions.

By encasing the targeting peptide cysteine–arginine–glutamic acid–lysine–alanine (CREKA) with two different amphiphilic molecules containing Gd, Yoo et al. [93] developed fibrin-binding peptide amphiphile micelles (PAMs). These molecules are the chelator diethylenetriaminepentaacetic acid (DTPA) as DTPA-bis(stearyl amide) and 1,2-distearoyl-sn-glycero-3-phosphoethanolamine-N-(PEG)-2000)-DTPA as (DSPE-PEG2000-DTPA). Using magnetic and optical imaging, an atherosclerotic mouse model was assessed for safety, contrast enhancement, and clot-binding properties in vitro. The fibrin specificity provided by the peptide ligand was demonstrated by the outcomes of optical and in vivo imaging investigations of the heart and aorta. According to biodistribution studies, all micelles were eliminated by the reticuloendothelial system and renal clearance. These studies demonstrated the efficaciousness of molecular imaging in vitro as opposed to in vivo for site-specificity and offered a platform for the theranostic application of contrast-enhancing agents to detect thrombosis [93].

To determine the potential of inhibitors aimed at cholesterol uptake in cultured Caco-2 cells, Kirana et al [94] compared the effectiveness of naturally occurring micelles derived from pig bile to artificially developed micelles. Pig bile was a practical and affordable source of micelles for cellular uptake and cholesterol micelle solubility assays and an effective way to screen possible agents that lower cholesterol [94].

Various macrophage-specific surface receptors, such as the mannose, folate, and scavenger receptors (SRs), can be affixed to micelles for efficient targeting. For instance, pH-responsive polymeric micelles with siRNA and mannose receptor-CD206 decoration were created by Yu et al. [95]. The micelles exhibited a 4-fold increase in siRNA delivery to macrophages and a knockdown of the model gene in macrophages of 87 ± 10% [95]. On a different note, monocyte-targeting micelles that bind to MCP-1’s CCR2-chemokine receptor have been described by Hyatt et al. [96]. These micelles attach themselves to macrophages and monocytes, labeling the atherosclerotic aortas in mice according to the degree of lesions [96].

Chin et al. [97] created multimodal theranostic peptide amphiphile micelles (PAMs) that bind to monocytes, inhibit collagenase and enhance the visualization of MRI images. These multimodal micelles contained a collagenase-cleaving peptide to thicken fibrous caps, a chelator of gadolinium (Gd), and diethylenetriaminepentaacetic acid (DTPA) as a contrast agent for (MRI) of plaques, and MCP-1 as the ligand to target monocytes. The multimodal PAMs effectively accumulated in lesions, markedly thickened the fibrous cap, and detected plaques in the aorta of ApoE^−/−^ mice in real time [97].

Less cholesterol and thicker enveloping caps on plaques make them stable and less likely to cause significant cardiac events. On the other hand, plaques that have large lipid cores, rich inflammatory macrophages and foam cells, and distinctively thin fibrous caps are brittle and susceptible. Two types of targeting micelles were compared by Kuo et al. [98]: VCAM-1 targeting micelles (which contained the VHPKQHR peptide) and fibrin targeting micelles (which contained the REKA peptide). These two micelles contained negatively charged miRNA inhibitors, which block dysregulated miRNAs in diseased cells linked to atherosclerosis. Next, using mouse macrophages and human aortic endothelial cells (HAECs), the in vitro effectiveness and efficiency of atherosclerotic lesion-specific targeting were investigated independently. The outcomes demonstrated that miRNA inhibitors could be transported and delivered into atherosclerotic lesions using fibrin-targeting and VCAM-1-targeting micelles. Collagen, elastin, and proteoglycans are components of the extracellular matrix in plaques that aid in their stabilization. In addition, there is a strong correlation between the characteristics of aortic plaques and the serum levels of MMPs, suggesting that MMPs may be a target for atherosclerosis. Reducing the activity of metalloproteinases like MMP-2, MMP-9, and MMP-12 would stabilize the fibrous cap because MMPs hasten the matrix’s breakdown. A glycoprotein known as extracellular matrix metalloproteinase inducer (EMMPRIN, CD147) controls the expression of MMP in monocytes, macrophages, and other cell types [98].

Alternatively, lipid micelles with an MMP-targeting peptide were created by Nguyen et al. [99], and it was discovered that these micelles colocalized with MMP and infiltrated macrophages. It has been common practice to create MMP-responsive polymer micelles for the site-specific release of pharmaceuticals using the MMP-2/9 cleavable peptide GPVGLIGK-NH2. To treat atherosclerosis, various ROS-responsive micelles have been created and studied; most of these micelles are conjugated by ROS-responsive linkers between hydrophilic and hydrophobic polymers. To design micelles, thioether groups and thioketal linkages are representative ROS-responsive linkers. High ROS concentrations cause these ROS-responsive linkers to break down and transition from hydrophobic to hydrophilic, breaking micelles and releasing drugs [99].

Gupta and colleagues [100] synthesized an ABC triblock polymer and assembled it into micelles with a temperature-responsive unit on the outer corona and propylene sulfide (PPS), a ROS-responsive unit, in the hydrophobic core. The micelles decreased ROS-mediated nonspecific damage to normal cells in vitro and exhibited ROS-responsive drug release characteristics. ROS-responsive micelles injected subcutaneously can extend the model drug’s 14-day local release [100].

Using the anti-inflammatory drug celastrol in the core, Allen et al. [101] created ROS-responsive nanocarriers called poly(ethylene glycol)-b-poly (propylene sulfide) (PEG-b-PPS) micelles. Celastrol-loaded PEG-b-PPS micelles successfully reduced the plaque area of Ldlr^−/−^ mice by significantly reducing cytotoxicity, improving anti-inflammatory efficacy, and drastically reducing the number of inflammatory monocytes and neutrophils in atherosclerotic plaques when compared to free celastrols.

The fibrous caps of atherosclerotic plaques develop microcalcifications 5 µm-100 µm, primarily composed of hydroxyapatite (HA, Ca_5_(PO_4_)_3_(OH)), which can cause plaque rupture because of the loss of compliance and elasticity. In the end, arterial occlusion and embolization brought on by plaque rupture can result in ischemic events like myocardial infarctions and strokes. Sadly, the low signal-to-noise ratio and invasive techniques used by current imaging technologies to identify calcifications increase the risk of arterial dissection. In this regard, a 12-amino acid HA-binding peptide (HABP) [SVSVGMKPSPRP] is used by Chin et al. [102] to create a novel fluorescently-labeled peptide amphiphile micelle (PAM) that targets and detects atherosclerotic calcification (HA PAM). According to the findings, HA PAMs can effectively target HA microcrystals in vitro with a strong binding affinity (KD = 6.26 1.2 mM). 

In preclinical research, engineered micelles have greatly improved atherosclerosis diagnosis and treatment. Various targeting elements, such as peptides, antibodies, and ROS-responsive linkers, have been used to improve the targeting efficacy of micelles in various atherosclerotic plaque components. Targeting micelles have been loaded with anti-inflammatory or antiangiogenic drugs, peptides, and genes to enhance the treatment of atherosclerosis. These preclinical studies have produced encouraging results, such as improved delivery efficacy, longer circulation times, reduced levels of proinflammatory cytokines, and reduced aortic plaque area [80,103].

Table 2 presents a summative overview of the most important treatments for cardiovascular diseases based on micelles.

## 4. Polymeric Nanoparticles

### 4.1. Types of Polymer-Based Nanoformulations and Their Relevant Features for CVDs

The most common class of materials used to create nanoparticles is polymers. Polymeric nanoparticles can be adjusted to control their hydrophobicity, degradability, and possible reabsorption within the body by controlling their synthesis conditions. Moreover, polymers are widely used as functionalization moieties, given their lower toxicity compared to metals and their availability of chemically active sites [104].

Polymers can be natural or synthetic. Natural polymers include starches, cellulose, latex, chitosan, gelatin, alginate, and proteins. Polymeric biodegradable nano-drug delivery systems are also widely produced using synthetic polymers, such as polylactic acid (PLA), polyglycolic acid, poly lactic-co-glycolic acid (PLGA), polyvinyl imine, polycaprolactone, and polyvinyl alcohol [27,105,106].

Typically, various techniques, including dissolution, encapsulation, embedding, and covalent attachment, are used to integrate drugs into polymer nanoparticles. The drug delivery capabilities of polymer NPs vary depending on the positions and patterns of drug combinations with NPs. Among the polymer NPs, the biodegradable poly(lactic-co-glycolic acid) (PLGA) is the most prevalent. Drug release can be regulated by packing drugs in PLGA NPs using the emulsified solvent diffusion method, which has been shown to mediate inflammatory cell recruitment and inhibit atherosclerotic plaque instability [81]. The FDA has approved polymeric nanoparticles made of polylactic acid (PLA), polyglycolic acid (PGA), and poly lactic-co-glycolic acid (PLGA). A copolymer of PLA and PGA, PLGA, is being investigated as a potential drug delivery mechanism for incurable illnesses like CVD [19].

Nanospheres or nanocapsules are typical forms of polymeric nanoparticles. Unlike drugs, nanospheres, or other solid particles inserted into a polymeric matrix, the therapeutic component in nanocapsules is encapsulated within a polymeric capsule shell. Generally speaking, they are less expensive and easier to produce and scale up than liposomes with longer stability profiles. When treating conditions like cancer, neurological illnesses, cardiovascular diseases, etc., these nanoparticles deliver the drug to a precise location and at a precise dosage [27,105].

### 4.2. Polymeric Nanoparticles—Drug/Gene Delivery Systems

Polymer nanoparticles have been used as drug carriers in developing nanosystems to treat atherosclerosis. Novel and improved therapeutic alternatives are needed to diagnose atherosclerosis and reduce the risk of major cardiovascular complications in patients who have received standard care [105].

According to Al Meslmani et al. [107], immobilizing PLGA nanoparticles on polytetrafluoroethylene was a good material for developing vascular grafts. Additionally, Ahadian et al. [108] described the procedure for combining polymers with nanomaterials for cardiovascular applications, developing a promising polyester and carbon nanotube scaffold.

Insulin-like growth factor (IGF)-1-complexed PLGA nanoparticles (PLGA-IGF-1 NPs) have been proposed by Chang et al. [109] to provide early cardioprotection following acute MI, enhance IGF-1 retention, and induce Akt phosphorylation through intramyocardial injection. To maintain the biological activity of IGF-1, three distinct sizes of PLGA particles (60 nm, 200 nm, and 1 μm) were produced and then complexed with IGF-1 via electrostatic force. Following MI, they immediately administered PLGA-IGF-1 NPs into the heart. Compared with the other two larger particles, the 60 nm-sized PLGA-IGF-1 NPs transported more IGF-1 and triggered greater Akt activation in cultured cardiomyocytes. Additionally, in a dose-dependent way, PLGA-IGF-1 NPs stopped doxorubicin-induced cardiomyocyte death and extended Akt activation in cardiomyocytes for up to 24 h. At 2, 6, 8, and 24 h, the PLGA-IGF-1 NP treatment considerably kept more IGF-1 in the myocardium than the IGF-1 alone treatment. Only hearts receiving PLGA-IGF-1 NP treatment showed signs of Akt phosphorylation in cardiomyocytes 24 h after MI; hearts receiving PBS, IGF-1, or PLGA NP injection did not exhibit this phenomenon. Significantly, 21 days following experimental MI in mice, a single intramyocardial injection of PLGA-IGF-1 NPs was enough to prevent cardiomyocyte death (*p* < 0.001), decrease infarct size (*p* < 0.05), and enhance left ventricle ejection fraction (*p* < 0.01) [109,110].

Ren et al. [111] decided to investigate the efficacy and underlying mechanism of miR-30b-5p-loaded PEG-PLGA nanoparticles (NPs) to treat heart failure. The impact of miR-30b-5p NPs on cardiac structure and function was evaluated by TUNEL staining, HE/Masson staining, immunofluorescence, and echocardiography. Dual luciferase reporter assay and RT-PCR were used to investigate the mechanism of miR-30b-5p therapy on heart failure, while Western blotting and RT-PCR were used to assess the effects of NPs on the expression of factors linked to cardiac hypertrophy and inflammation. PEG-PLGA NPs ranged in size from 200 to 300 nm and had a negative zeta potential. The loading levels varied. The NPs for miR-30b-5p had a high mean entrapment efficiency of 81.8 ± 2.1%, and their release rate was over 90% after 5 days. Distribution studies revealed that NPs were mostly found in the heart and that they protected cardiac function and myocardial damage. In contrast to a rat model of heart failure and the miR-30b-5p-non-loaede NP groups, there was a significant decrease in the production of inflammatory factors (IL-1β, IL-6) and cardiac hypertrophy markers (ANP, BNPβ-MHC) [111]. According to Ma et al.’s [112] research, stem cell transplantation is a great therapeutic option for myocardial infarction. Melatonin was used, along with the preparation of poly(lactide-co-glycolide)-monomethoxy-poly(polyethylene glycol) (PLGA-mPEG) nanoparticles, to shield the cells from oxidative damage. Adipose-derived mesenchymal stem cells were exposed to melatonin-encapsulated PLGA-mPEG nanoparticles, or melatonin nanoparticles (Mel-NPs), in vitro and in vivo. Combining stem cell transplantation with Mel-NPs could be a promising treatment approach for myocardial infarction [112].

The field of biomedicine has shown a great deal of interest in natural polysaccharides. Polysaccharides are long chains of various mono-saccharides, such as alginate (algae), pectin, guar gum (plant), dextran, xanthan gum (microbe), and chondroitin (animal) [113]. Their structure and property diversity are influenced by their wide range of molecular weights, numerous reactive groups, and varied chemical compositions. These naturally occurring biomaterials are biocompatible, hydrophilic, biodegradable, and non-toxic. Interestingly, most natural polysaccharides have hydrophilic groups like amino, hydroxyl, and carboxyl groups. Considerable attention has been focused on polysaccharides and their derivatives in recent years due to the possibility of using them as nano-drug delivery systems [114].

Polysaccharides play various physiological roles, including adhesion and cell signaling. They also serve as therapeutic agents. Polysaccharides can identify the biological molecules involved in the atherothrombotic process. Targets of the polysaccharide-ride interaction in the development of atherothrombosis include fibrin, antithrombin, macrophage receptors, selectins, and cholesterol. Important polysaccharide-based nanosystems frequently used to treat antithrombotic diseases include guar gum, locust bean gum, fucoidan, chitosan, cyclodextrin, heparin, and hyaluronic acid [114].

Cardiovascular illnesses have consistently been linked to necroptosis, a unique type of programmed cell death characterized by cell enlargement, plasma membrane rupture, and organelle failure. The main ingredient in sugarcane leaves with a heart-protective effect is sugarcane leaf polysaccharide (SLP). Still unknown, nonetheless, are the benefits of SLP and the underlying mechanisms in myocardial ischemia-reperfusion (MI/R). This study looked at both in vitro and in vivo settings to examine the protective effects of SLP on MI/R damage. In vitro tests utilizing tertiary butyl hydrogen peroxide (TBHP)-stimulated H9c2 cells and in vivo tests using Sprague Dawley rats were used to evaluate the protective effects of SLP against MI/R injury. By preventing necroptosis and oxidative stress, SLP dramatically prevented TBHP-induced H9c2 cell death in vitro. SLP used the Nrf2/HO-1 pathway to stimulate antioxidant activity. By reducing the phosphorylation of RIP1, RIP3, and MLKL in TBHP-stimulated H9c2 cells, SLP inhibited necroptosis. SLP reduced the myocardial infarct area, increased myeloperoxidase and superoxide dismutase levels, and decreased interleukin-6, tumor necrosis factor-α, and malondialdehyde levels in vivo to attenuate MI/R injury [115].

Polysaccharide-based nanosystems are the standard approach for targeting particular cardiovascular pathologies, such as atherothrombotic diseases. By delivering a glycolysis inhibitor, polysaccharide (sugar) nanoparticles normalized vasculature and activated macrophage metabolism, reducing inflammation associated with atherosclerotic plaque in murine models. Other non-immune-mediated strategies use polymeric nanodelivery to stimulate antioxidant responses by the biochemical machinery [25,116]. In their research, Luong-Van et al. [117] noted that heparin was integrated into poly (ε-caprolactone) nanofibers to enable regulated heparin release to the locations of vascular injury during the treatment of intimal hyperplasia.

To passively target the ischemic tissue, the drug vehicle must be administered soon after the infarction occurs and have particular physicochemical properties regarding size, shape, and surface modification. The optimal time to treat MI is 24 h after it occurs when cell apoptosis is at its highest. Notably, prolonged circulation of some growth factors can have unfavorable effects; therefore, it is necessary to release them immediately [118]. Insulin-like growth factor-1 (IGF-I), for example, prevents cardiomyocytes from dying, but overexpressing can hinder the recovery of cardiac function. To establish the required electrostatic binding and preserve IGF-I, negatively charged IGF-I was thus conjugated to negatively charged PLGA NPs of various sizes after being modified with positively charged PEI (poly ethylenimine). In contrast to the free administration of IGF-I, PLGA NPs were limited to the injured tissue. After 24 h, the 60 nm NPs exhibited the highest IGF-I binding affinity and retention, preserving cardiac function by decreasing the infarct size and averting ventricular remodeling [119].

Cardiomyocyte apoptosis can be prevented in the infarcted heart by reducing chronic inflammation. The passive delivery of pitavastatin in PLGA NPs effectively triggered the protein kinase B/phosphatidylinositol 3-kinase pathway, preventing cell death in hypoxic cells and subsequent reoxygenation. Another component of an ischemic tissue’s niche microenvironment is ROS, which are produced during I/R injuries. In addition to causing oxidative stress during inflammation, nicotinamide adenine dinucleotide phosphate (NADPH) oxidases are also increased in MI. As a result, lysine-based nanoparticles (NPs) contain siRNA that targets the enzyme NADPH oxidase 2 (NOX2) [120]. Local delivery reduced ROS production and inhibited neointimal hyperplasia in an in vivo atherosclerotic model. Additionally, the placental growth factor was delivered to the ischemic myocardium, and its release was sustained by alginate/chitosan NPs, which also prevented its degradation. Increased capillary and arteriole densities, increased release of the anti-inflammatory cytokine IL-10, and decreased release of the proinflammatory cytokines TNF-α and IL-6 were all observed after treatment with placental growth factor-loaded NPs [121].

Heart damage is acknowledged as a primary cause of multiple organ failure linked to sepsis and as a major contributor to septic shock. Recent research indicates that controlling the inflammatory response and intramyocardial oxidative stress has bright futures. An anti-inflammatory and antioxidant basic fibroblast growth factor (bFGF) is demonstrated. Li et al [122] aimed to deliver bFGF (bFGF-RBC/NP) for sepsis-induced cardiac damage using red blood cell membrane-camouflaged poly (lactide-co-glycolide) nanoparticles. Cardiomyocytes could be shielded from oxidative and inflammatory damage by bFGF-RBC/NP, according to the in vitro tests. Moreover, information from in vivo studies was used to confirm the antioxidant and anti-inflammatory qualities of bFGF-RBC/NP against heart damage. The results showed the therapeutic effects of bFGF-RBC/NP in managing myocardial dysfunction by using it to treat sepsis-induced cardiac damage. This work offers a new approach to treating and preventing heart damage in sepsis [122].

Adipose-derived stem cells (ASCs) have been shown to internalize PLGA NPs conjugated to simvastatin in vitro. Additionally, ASCs have been shown to express pro-angiogenic and anti-apoptotic factors, enhance their differentiation towards smooth muscle and endothelial cells, and promote ASC migration in response to drug release from slowly degrading PLGA NPs. These findings were translated in vivo in a murine model of MI, wherein stem cells treated with simvastatin-NPs were attracted to and retained in the ischemic region, resulting in enhanced cardiac function and infarcted heart regeneration. Additionally, 270 nm core-shell NPs with a Pluronic F-127 shell and a lecithin/VEGF core were made for MI heart regeneration. The nanoparticles demonstrated the ability to continuously release VEGF for a duration of 42 days. The NPs greatly increased capillary density and enhanced cardiac function in vivo. Adding Capryol 90, a non-ionic water-insoluble surfactant, NPs gelation was accomplished, and a gel network containing NPs loaded with VEGF was formed. Compared to the loaded NPs, the hydrogel was found to have a greater impact on restoring cardiac function, indicating its benefit in enhancing NP localization within the ischemic heart [123].

Furthermore, ASCs, mesenchymal stem cells-derived secretome, and cardiac stem cells (CSC) were all encapsulated using different hydrogels made from naphthalene molecules and β-galactose caged nitric oxide (NO) donors (NapFF-NO), gelatin methacrylate (GelMA) complexed with nanosilicates, and agarose supplemented with integrin-binding proteins [124]. NapFF-NO hydrogel increased the survival of stem cells and improved cardiac function when NO stimulated pro-angiogenic factors. Additionally, the secretome-rich hydrogel of GelMA/nanosilicates exhibited pro-angiogenic and cardioprotective properties, improving the survival of cardiomyocytes following hypoxia-induced apoptosis.

To accelerate the vascularization of decellularized buffalo bovine jugular vein scaffolds, self-assembled polymeric NPs made of heparin and chitosan were immobilized to the nanofibers. These NPs were created for the sustained release of VEGF [125]. In an in vivo mouse subcutaneous implantation model, the NPs effectively increased fibroblast infiltration, ECM production, and new capillary formation, demonstrating encouraging outcomes for potential MI applications in the future. Furthermore, it was discovered that bilayered NPs with a PLGA core and PLLA shell could cause the sequential release of platelet-derived growth factor after simultaneously releasing VEGF and bFGF. They were discovered to stimulate angiogenesis in an ex vivo rat aortic ring experiment after being incorporated in a fibrin matrix, indicating their possible use in MI repair scaffolds [119,126].

Wang and colleagues employed an injectable hydrogel that was sensitive to H_2_O_2_ and fortified with the self-assembled nanodrug tanshinone IIA. To guarantee their entrapment within the hydrogel via chemical cross-linking, the NPs were coated with polydopamine. In a rat model of MI, the hydrogel slowly broke down after injection, allowing the drug to be released over time inside the infarct [127]. This led to an increase in the LV ejection fraction, a decrease in the infarct size, and an inhibition of inflammatory factors. A different injectable hydrogel with shear-thinning characteristics was created to locate polymeric NPs loaded with miRNA. One microRNA that has already shown promise in treating CVDs is miR-199a-3p [128]. As a result, it became trapped in poly (9,9-dioctylfluorene-alt-benzothiadiazole)/PEG core-shell nanoparticles. The NPs demonstrated significant promise in stimulating endothelial and cardiac cell proliferation and angiogenesis under hypoxic conditions. The NPs/hydrogel system successfully increased capillary density in the infarct border zone and improved cardiac function in vivo [129].

To functionalize conductive engineered cardiac patches (ECP) as alternatives to infarcted tissue, other researchers concentrated on synthesizing polymeric NPs because they can activate living cardiomyocytes and bridge electrical signals from the healthy myocardium across scar tissue. Using a cross-linker inspired by mussels, conductive nanoparticles (NPs) composed of gelatin methacrylate/polypyrrole (GelMA/Ppy) were cross-linked onto an electrospun GelMA/polycaprolactone nanofibrous membrane [130]. This biocompatible ECP was implanted in a rat model of MI, and after four weeks, it improved cardiac function and encouraged revascularization, healing the infarcted myocardium [131].

Wang et al. created Ppy NPs and used a cross-linker inspired by mussels to incorporate them into a GelMA/PEG cryogel to achieve the best mechanical and superelastic properties for the ECP. The GelMA/PEG NPs were demonstrated to migrate from the ECP, fuse to the surface of cardiomyocytes, and encourage their synchronous contraction, which enhanced cardiac function in an in vivo rat MI model. Overall, polymeric NPs have been demonstrated to be suitable DDS for administering medications and/or growth factors in various scaffolds for the healing of the damaged heart following myocardial infarction [132].

Table 3 presents the most important types of polymeric nanoparticles and their medical applications in cardiovascular disease.

### 4.3. Dendrimers

The unique branched, tree-like molecular structure of dendritic polymers features numerous void spaces internally and a multitude of functional groups on the outer surface. The number of these functional groups is precisely determined for each generation of the polymer resulting in practically monodisperse nanoparticles. This architecture proves highly beneficial for biomedical applications. The voids can host various therapeutic and imaging agents, while the outer functional groups enhance the nanovehicle’s solubility in biological fluids. Additionally, these groups can serve as anchoring points for covalent bonding with other biologically active molecules or targeting ligands. Due to their unique properties, dendritic polymers have been explored for various applications in the diagnosis and treatment of cardiovascular diseases, as follows.

a. Nano-drug delivery systems: In the context of cardiovascular diseases, dendrimers can be tailored to deliver anti-inflammatory, anti-thrombotic, or vasodilator drugs directly to the affected sites, minimizing systemic side effects.

A nice application for the delivery of the thrombus dissolving enzyme nattokinase (NT) was reported by S-F Zhang et al. [138]. They used as the nanocarrier a pegylated peptide based on different generations (G2–G4) of dendritic polyglutamic acid. The dendritic architecture was constructed using PEG difunctionalized at both ends with azide moieties and 4-pentynoic acid in a click-chemistry manner for the covalent attachment of the first two rests of glutamic acid. Next, the following dendritic generations of the peptide nanocarrier were grown in the usual stepwise manner using the well-documented peptide synthesis. The authors took advantage of the attractive electrostatic interactions to encapsulate the positively charged NK into the negatively charged Glu_n_–PEG–Glu_n_ dendritic peptide nanovehicle, thereby preserving the enzyme activity and avoiding its fast premature biodegradation. Moreover, the nanoencapsulation extended the enzyme circulation time enabling enhanced thrombus dissolution.

Nitric oxide (NO) released by the vascular endothelium plays a direct role in vascular smooth muscle relaxation, thereby regulating vascular tone [139]. Many processes contributing to ischemia/reperfusion (I/R) injury are mediated by NO. However, the therapeutic effects are highly sensitive to the concentration of NO. When administered at low concentrations, NO has been shown to improve cardiomyocyte function [139]. In contrast, high levels of NO induce inflammatory processes, impair mitochondrial respiration, and eventually lead to cardiomyocyte death [14,140]. This delicate balance presents significant challenges, limiting clinical applications due to the difficulty of quantifying a standard I/R injury protocol for gaseous NO administration. Within the body, the primary carriers of NO are *S*-nitrosothiols (RSNO), which release NO either through direct transnitrosation with proteins or via decomposition initiated by enzymatic metal centers [141]. Stasko et al. [142] designed NO carriers based on G4 poly(amidoamine) (PAMAM) dendrimers with 64 NH2 groups on the outer surface (G4-NH2). These 64 amino groups were modified with either *N*-acetyl-D,L-penicillamine (NAP) or *N*-acetyl-L-cysteine (NACys). Subsequently, the terminal thiol groups of the resulting two G4 dendrimers were converted to *S*-nitrosothiol groups upon treatment with sodium nitrite solutions, yielding G64-SNAP and G64-NACCysNO as NO donors, respectively. The authors evaluated the ability of G4-SNAP to inhibit thrombin-mediated platelet aggregation. They compared the degree of platelet aggregation inhibition achieved using the dendrimer NO donor to that obtained with the small SNAP molecule at equivalent nitrosothiol concentrations (25 μM). While the small SNAP molecule exhibited 17% inhibition of platelet aggregation, the dendrimer NO donor (G64-SNAP) resulted in 62% inhibition. The specific dendritic molecular architecture allows for more precise control over the amount of released NO.

A widely prescribed medication for lowering cholesterol levels and preventing atherosclerotic plaque formation is simvastatin (SMV). However, its therapeutic efficacy is limited by low solubility, weak bioavailability, and irregular absorption. To improve the pharmacokinetic profile of SMV, Kulhari et al. used several types of PAMAM dendrimers, namely, PAMAM–NH_2_, PAMAM–OH, and PAMAM–PEG, as delivery vehicles [143]. The complexes SMV–dendrimers showed a controlled release profile in vitro studies. While genuine SMV was released in 5 h from the complex with the pegylated dendrimer SMV/PEG-PAMAM, the hypolipidemic drug was released up to 5 days later. The other non-pegylated nanovehicles released the drug up to 24 h later. The authors concluded that pegylated dendrimer formulations gave the best outcomes in terms of solubility enhancement, better dissolution, increased biocompatibility, slower release rate, and more stability on storage in dark as compared to pure SMV or non-pegylated dendrimers. Nifedipine is a calcium channel blocker used in anti-hypertensive and anti-anginal medication. In order to improve its solubility and bioavailability properties, Devarakonda et al. [144] used PAMAM dendrimers to study the effect of the dendrimer generation and pH on the solubility of nifedipine. They used both amine and ester-terminated dendrimers. At each pH value the solubility of nifedipine encapsulated in dendrimers possessing the same number of functional groups was higher for the ester-terminated dendrimers. At pH 7, the nifedipine solubility decreased in the following order: G2.5 > G3 > G1.5 > G2 ≥ G0.5 > G1 > G0. The authors concluded that the observed solubility trend was due to the degree of changes in the ionization of the basic amino groups. A decrease in the protonation of dendritic amines at increased pH values results in the formation of hydrogen bridges between the tertiary amines within the dendrimer cavity and the nifedipine drug.

A different study described the creation of a poly(amidoamine) (PAMAM) dendrimer-based drug delivery system that combines the delivery of hydrochlorothiazide (HCTZ) and ramipril (RAPL). The drugs HCTZ (a diuretic) and RAPL (an antihypertensive) are used to treat high blood pressure. The amine-terminated dendrimer increased the RAPL solubility 4.91 times at 0.8% (*w*/*v*) dendrimer concentration, while the carboxy-terminated dendrimer was the best for HCTZ solubilization (3.72-fold). The dissolution rate was faster when the new formulations were compared to the free drugs, whereas the dissolution patterns were similar for hybrid formulations and single drug-loaded dendrimers. According to the authors, the formulation strategy showed promise for application, and further work is being conducted to advance this technology [51,145].

b. Imaging Agents: Dendritic polymers can be functionalized with imaging probes such as fluorophores, radioisotopes, or magnetic resonance imaging (MRI) contrast agents. These modified dendrimers can serve as targeted imaging agents to visualize vascular inflammation, atherosclerotic plaques, or myocardial infarction, allowing for the early diagnosis and monitoring of cardiovascular diseases.

Fu et al. [146] developed novel dendritic iodinated contrast agents with PEG core for CT microvasculature imaging. They constructed a “bow-tie” dendrimer by growing successive lysine-based dendron generations from both ends of the hydrophilic and biocompatible PEG core. The free terminal amino groups of the bow-tie dendrimer were used as anchoring points for the covalent attachment of the highly soluble and reactive triiodophthalimide moieties as the contrast agent. These innovative dendritic nanoparticles showed excellent characteristics in terms of high solubility, high iodine content, high molecular weight, and circulation time extension, which was prolonged up to 35 min as compared to smaller molecular contrast media that enable much shorter vascular enhancement lasting for only 5 min in a rat model. These outstanding properties recommend them as excellent candidates for potential applications in angiography and quantitative imagistic exploration of ischemic cardiac injury like myocardial infarction (MI).

Ardestani et al. designed a dendrimer glyco-conjugate (DGC) for technetium 99 m radionuclide accurately diagnosis of myocardial infarctions at early stages [147]. The dendritic nano-agent comprises a PEG 600 diacid core conjugated at both ends, first with citric acid and then with glucosamine moieties, imparting both biocompatibility and biodegradability to the entire assembly. Moreover, the capacity of citric acid to complex with ^99m^Tc is a crucial feature for radionuclide MI diagnosis. The authors tested the dendritic imaging agent in two rabbit models, with and without infarction. Using single-photon emission computed tomography (SPECT) and dynamic planar imaging they clearly demonstrated accumulation of ^99m^Tc-DGC complex in myocardial. In vitro XTT ((sodium 3′-[1-(phenylaminocarbonyl)-3,4-tetrazolium]-bis (4-methoxy6-nitro) benzene sulfonic acid hydrate) cell viability assay carried out on HEK-293 cell line showed lack of toxicity up to 8 mg/mL concentration.

c. Gene Delivery: Dendritic polymers can be used as non-viral vectors for gene delivery to cardiac or vascular cells. By encapsulating or complexing with nucleic acids, dendrimers can facilitate targeted gene transfer, gene silencing, or gene editing, opening up new avenues for gene therapy in cardiovascular diseases.

Angiotensin II is an octa-peptide hormone overexpressed following MI. Elevated levels of this hormone cause cardiomyocyte death and hypertrophy, vascular smooth muscle growth, and fibrosis leading to adverse cardiac remodeling, progressive ventricular dysfunction, and eventually heart failure [148]. The Angiotensine II type I receptor (AT1R) is the key mediator of these adverse effects and therefore silencing gene expression of AT1R using small interfering RNA (siRNA) is one of the best choices in order to restore blood pressure, prevent fibrosis and improve cardiac function after MI. However, the internalization of small RNA molecules is significantly limited in cardiomyocytes due to their non-phagocytic nature. Liu et al. [149] developed a non-viral, non-cytotoxic siRNA delivery system specifically for cardiac tissue. They engineered a ‘tadpole’-like dendrimer with the following structure: a dendritic PAMAM head for siRNA complexation and a cell-penetrating peptide (CPP) i.e., Arg_9_ or TAT tail to enhance cellular uptake. These two components were linked by a PEG linker through biocompatible disulfide bridges [149]. When tested in vivo, the designed tadpole like dendrimer prevented the increase in AT1R levels and showed better clinical outcomes in terms of cardiac function recovery compared to usually used saline injection in ischemia reperfusion injury.

In order to mitigate cardiomyocyte apoptosis in hypoxic cardiac cells, Xue et al. [150] developed a nanovector for targeted delivery of microRNA-1 inhibitor at the infarct site. Micro-RNA-1 acts by expression of apoptotic genes; consequently, its inhibition reduces apoptosis [151]. Taking advantage of the overexpression of AT1 receptors in infarcted heart, the authors designed a PEG modified dendri-grafted poly-l-lysine (DGL) dendrimer conjugated to AT1 targeting ligand (AT1–PEG–DGL). The complex between the anti-miR-1 antisense oligonucleotide (AMO-1) and the nanovector (AT-1–PEG–DGL@AMO-1) was administered by IV injection in a mouse MI model. In vivo imaging proved rapid accumulation of the nanovehicle in the MI heart while the myocardial infarct size was reduced by 64.1% as compared with that in the MI control group upon a single IV injection [152].

## 5. Conclusions

In summary, organic nanoparticles have demonstrated several benefits over traditional therapies in diagnosing and treating CVDs, showing a great deal of promise in stimulating the regeneration of damaged cardiac cells. Due to their many advantages, such as biocompatibility, drug encapsulation efficiency, targeted gene therapy, and their use in medical imaging, especially for atherosclerosis, myocardial infarction, and other CVDs, organic nanoparticles may represent an improved treatment alternative. According to the findings of the recent literature, organic nanoparticles of a smaller size are recommended for drug delivery systems due to their capacity to penetrate the capillaries more readily.

This paper discussed organic nanoparticles, mainly dividing them into lipid- and polymeric-based materials according to their chemical composition. Polymeric NPs hold great promise as a delivery system to overcome the shortcomings of existing methods for diagnosing and treating various CVDs. To treat stent-related complications non-invasively, stents coated with polymeric material containing dispersed or encapsulated drugs have been developed. Moreover, lipid-based nanoparticles can be used with polymer-coated stents or as stand-alone drug delivery systems, with particular advances being noted in developing liposome- and micelle-based therapeutics. In addition, chemical species have been able to reside at the focal core of a dendrimer due to the unique nanoscale architecture produced by extensive branching, which also allows for various interactions between other dendrimers and the surrounding molecular environment.

Regarding the usage of organic nanoparticles, there are still several challenges to overcome, such as the stability of nanoparticles in the human body and the control of their size and shape. Moreover, toxicity studies on nanomedicine, a novel form of therapy, have frequently been disregarded in recent decades. There is currently no standard to assess and classify the hazardous levels of different organic nanoparticles, despite growing interest in their safety. Standardizing a comprehensive toxicology protocol is therefore necessary. Particular consideration must be given to the biological fate of the materials and their cellular toxicity when applying them to the heart, particularly when cardioprotection aims to preserve cells.

To conclude, the drug delivery strategies based on organic nanoparticles for treating cardiac diseases were reviewed in this paper, focusing on the limitations and recent developments regarding the use of lipids, polymers, and micelles as drug delivery tools in the future. It is believed that organic NPs will be widely used in clinics to improve patients’ quality of life, as research on these materials is constantly expanding and reshaping the field of nanomedicine. Nonetheless, existing research should be deepened, adding the clinical testing dimension to the discussed nanoformulations before putting them on the market.

## Figures and Tables

**Table 1:** Liposome-based treatments for cardiovascular disease.

Type of Treatment	Medical Applications	References
Vesicles formulated with phospholipids and PEG-HS and istaroxime	Acute and chronic heart failure	[47]
Biomimetic liposomes	Anti-inflammatory treatment of MI	[61]
Liposome/DNA complexes immobilized on the stainless steel surface	Coronary restenosis	[62]
Liposome plasmid DNA complexes with TATp	Transfection of cardiomyocytes in the ischemic zone	[68]
Targeted liposomes loaded with ATP	Cardioprotective effect	[79]
Liposomes coated with polyethylene glycol with gadodiamide	Enhance MR angiography	[76]

**Table 2:** Micelle-based treatments for cardiovascular disease.

Type of Treatment	Medical Applications	References
Surface-modified polymeric micelles	Drug delivery and theranostic	[83]
PEG-based lipid micelle system	Delivery of anticoagulant drugs	[86]
Gd-encapsulated micelles functionalized with anti-CD36 antibodies	Identifying macrophages in human atherosclerotic aortic tissues	[87]
pH-responsive polymeric micelles with siRNA and mannose receptor-CD206 decoration	siRNA delivery to macrophages	[95]
Multimodal theranostic peptide amphiphile micelles	Enhancing the visualization of MRI	[97]
ROS-responsive micelles	Atherosclerosis treatment	[99]

**Table 3:** Polymeric nanoparticle-based treatments for cardiovascular disease.

Type of Treatment	Medical Applications	References
PLGA nanoparticles	Vascular grafts Drug delivery systems Myocardial infarction	[107,108,109]
Polysaccharide nanoparticles	Thrombolysis Reduction in inflammation associated with atherosclerotic plaque	[116,117,133,134,135,136,137]
Chitosan nanoparticles	Antiatherosclerosis properties Drug delivery systems	[117,136]
GelMA	Pro-angiogenic and cardioprotective properties Improvement in cardiac function Superelastic properties	[83,124,125]

## Data Availability

Not applicable.

## Abbreviations

ADM-2	Adrenomedullin-2
AMI	Acute myocardial infarction
ASCs	Adipose-derived stem cells
AT1R	Angiotensin II receptor type 1
ATP-L	ATP-liposomes
CAD	Coronary artery disease
CARPA	C activation related pseudoallergy
CREKA	Cysteine-arginine-glutamic acid lysine alanine
CSC	Cardiac stem cells
CVDs	Cardiovascular diseases
DDS	Drug delivery system
Doxil	Doxorubicin
DTPA	Diethylenetriaminepentaacetic acid
ECP	Engineered cardiac patches
ECS 304	Endothelial cells
GD	Gadolinium
GelMA/Ppy	Gelatin methacrylate/polypyrrole
HA	Hydroxyapatite
HABP	12-amino acid HA-binding peptide
HAECs	Human aortic endothelial cells
HCTZ	Hydrochlorothiazide
HSRs	Hypersensitivity responses
IGF-1	Insulin-like growth factor-1
IHD	Ischemic heart disease
KH	Krebs-Henseleit buffer
Mel	Melatonin
MI	Myocardial infarction
MRI	Magnetic resonance imaging
mTHPC	m-tetra(hydroxyphenyl)chlorin
NADPH	Nicotinamide adenine dinucleotide phosphate
Neu-LPs	Biomimetic liposomes
NK	Nattokinase
NO	Nitric oxide
NOX2	NADPH oxidase 2
NPs	Nanoparticles
PAMAM	Poly(amidoamine)
PAMs	Peptide amphiphile micelles
PCSK9	Proprotein convertase subtilisin–kexin type 9
PEG	Polyethylene glycol
PEI	Poly ethylenimine
PGA	Polyglycolic acid
PIGF	Placental growth factor
PLA	Polylactic acid
PLGA	Poly lactic-co-glycolic acid
PLGA-mPEG	Poly(lactide-co-glycolide)-monomethoxy-poly(polyethylene glycol)
PPS	Propylene sulfide
RAPL	Ramipril
ROS	Reactive oxygen species
siRNA	Small interfering ribonucleic acid
SMC	Smooth muscle cells
SRs	Scavenger receptors
TATp	Transactivating transcriptional activator peptide
t-PA	Tissue plasminogen activator
VEGF	Vascular endothelial growth factor
VSMCs	Vascular smooth muscle cells

## References

[1] Chen Y., Freedman N.D., Albert P.S., Huxley R.R., Shiels M.S., Withrow D.R., Spillane S., Powell-Wiley T.M., Berrington de González A. (2019). Association of Cardiovascular Disease With Premature Mortality in the United States. JAMA Cardiol..

[2] Khan M.A.B., Hashim M.J., Mustafa H., Baniyas M.Y., Al Suwaidi S.K.B.M., AlKatheeri R., Alblooshi F.M.K., Almatrooshi M.E.A.H., Alzaabi M.E.H., Al Darmaki R.S. (2020). Global epidemiology of ischemic heart disease: Results from the global burden of disease study. Cureus.

[3] Zhang L., Li Q., Han X., Wang S., Li P., Ding Y., Zhang T., Zhao J., Chen Y., Liu J. (2020). Associations of socioeconomic factors with cause-specific Mortality and burden of cardiovascular diseases: Findings from the vital registration in urban Shanghai, China, during 1974–2015. BMC Public Health.

[4] Masaebi F., Salehi M., Kazemi M., Vahabi N., Azizmohammad Looha M., Zayeri F. (2021). Trend analysis of disability adjusted life years due to cardiovascular diseases: Results from the global burden of disease study 2019. BMC Public Health.

[5] Flora G.D., Nayak M.K. (2019). A Brief Review of Cardiovascular Diseases, Associated Risk Factors and Current Treatment Regimes. Curr. Pharm. Des..

[6] Robinson J.G., Fox K.M., Bullano M.F., Grandy S., the SHIELD Study Group (2009). Atherosclerosis profile and incidence of cardiovascular events: A population-based survey. BMC Cardiovasc. Disord..

[7] Xu M., Zhang K., Song J. (2021). Targeted Therapy in Cardiovascular Disease: A Precision Therapy Era. Front. Pharmacol..

[8] Severino P., Amato A., Pucci M., Infusino F., Adamo F., Birtolo L.I., Netti L., Montefusco G., Chimenti C., Lavalle C. (2020). Ischemic Heart Disease Pathophysiology Paradigms Overview: From Plaque Activation to Microvascular Dysfunction. Int. J. Mol. Sci..

[9] Hu Q., Fang Z., Ge J., Li H. (2022). Nanotechnology for cardiovascular diseases. Innovation.

[10] Stanner S., Coe S., Frayn K.N. (2019). Cardiovascular Disease: Diet, Nutrition and Emerging Risk Factors.

[11] World Health Organization (2020). Hearts: Technical Package for Cardiovascular Disease Management in Primary Health Care.

[12] George C.E., Ramadas D., Norman G., Mukherjee D., Rao T. (2016). Barriers to cardiovascular disease risk reduction: Does physicians’ perspective matter?. Indian Heart J..

[13] Mukundan S., Ghaghada K.B., Badea C.T., Kao C.-Y., Hedlund L.W., Provenzale J.M., Johnson G.A., Chen E., Bellamkonda R.V., Annapragada A. (2006). A Liposomal Nanoscale Contrast Agent for Preclinical CT in Mice. Am. J. Roentgenol..

[14] Gupta P., Garcia E., Sarkar A., Kapoor S., Rafiq K., Chand H.S., Jayant R.D. (2019). Nanoparticle Based Treatment for Cardiovascular Diseases. Cardiovasc. Hematol. Disord. Drug Targets.

[15] Flores A.M., Ye J., Jarr K.-U., Hosseini-Nassab N., Smith B.R., Leeper N.J. (2019). Nanoparticle Therapy for Vascular Diseases. Arterioscler. Thromb. Vasc. Biol..

[16] Murthy S.K. (2007). Nanoparticles in modern medicine: State of the art and future challenges. Int. J. Nanomed..

[17] Salem S.S., Hammad E.N., Mohamed A.A., El-Dougdoug W. (2023). A comprehensive review of nanomaterials: Types, synthesis, characterization, and applications. Biointerface Res. Appl. Chem.

[18] Evers M.J.W., Du W., Yang Q., Kooijmans S.A.A., Vink A., van Steenbergen M., Vader P., de Jager S.C.A., Fuchs S.A., Mastrobattista E. (2022). Delivery of modified mRNA to damaged myocardium by systemic administration of lipid nanoparticles. J. Control. Release.

[19] Fan C., Joshi J., Li F., Xu B., Khan M., Yang J., Zhu W. (2020). Nanoparticle-Mediated Drug Delivery for Treatment of Ischemic Heart Disease. Front. Bioeng. Biotechnol..

[20] Chen H.-A., Ma Y.-H., Hsu T.-Y., Chen J.-P. (2020). Preparation of Peptide and Recombinant Tissue Plasminogen Activator Conjugated Poly(Lactic-Co-Glycolic Acid) (PLGA) Magnetic Nanoparticles for Dual Targeted Thrombolytic Therapy. Int. J. Mol. Sci..

[21] Chopra H., Bibi S., Mishra A.K., Tirth V., Yerramsetty S.V., Murali S.V., Ahmad S.U., Mohanta Y.K., Attia M.S., Algahtani A. (2022). Nanomaterials: A Promising Therapeutic Approach for Cardiovascular Diseases. J. Nanomater..

[22] Shen M., Wang Y., Hu F., Lv L., Chen K., Xing G. (2021). Thrombolytic Agents: Nanocarriers in Targeted Release. Molecules.

[23] Yu H., Palazzolo J.S., Zhou J., Hu Y., Niego B., Pan S., Ju Y., Wang T.Y., Lin Z., Hagemeyer C.E. (2022). Bioresponsive Polyphenol-Based Nanoparticles as Thrombolytic Drug Carriers. ACS Appl. Mater. Interfaces.

[24] Liu H., Pietersz G., Peter K., Wang X. (2022). Nanobiotechnology approaches for cardiovascular diseases: Site-specific targeting of drugs and nanoparticles for atherothrombosis. J. Nanobiotechnology.

[25] Smith B.R., Edelman E.R. (2023). Nanomedicines for cardiovascular disease. Nat. Cardiovasc. Res..

[26] Devries S., Rakel D. (2018). Chapter 26—Coronary Artery Disease. Integrative Medicine.

[27] Niculescu A.-G., Grumezescu A.M. (2021). Polymer-Based Nanosystems—A Versatile Delivery Approach. Materials.

[28] Kumar R., Mohapatra S.S., Ranjan S., Dasgupta N., Mishra R.K., Thomas S. (2019). Chapter 8—Lipid-Based Nanoparticles for Drug-Delivery Systems. Nanocarriers for Drug Delivery.

[29] Baccile N., Cuvier A.-S., Prévost S., Stevens C.V., Delbeke E., Berton J., Soetaert W., Van Bogaert I.N.A., Roelants S. (2016). Self-Assembly Mechanism of pH-Responsive Glycolipids: Micelles, Fibers, Vesicles, and Bilayers. Langmuir.

[30] Labonia C., Estape Senti M., Brans M., Snijders C., Sluijter J.P.G., Vader P. (2022). Modified mRNA delivery to the heart using Lipid Nanoparticles. Cardiovasc. Res..

[31] Bitounis D., Fanciullino R., Iliadis A., Ciccolini J. (2012). Optimizing druggability through liposomal formulations: New approaches to an old concept. Int. Sch. Res. Not..

[32] Bozzuto G., Molinari A. (2015). Liposomes as nanomedical devices. Int. J. Nanomed..

[33] Xu H., Li S., Liu Y.-S. (2022). Nanoparticles in the diagnosis and treatment of vascular aging and related diseases. Signal Transduct. Target. Ther..

[34] Mulder W.J., Strijkers G.J., van Tilborg G.A., Griffioen A.W., Nicolay K. (2006). Lipid-based nanoparticles for contrast-enhanced MRI and molecular imaging. NMR Biomed..

[35] Cruz-Samperio R., Hicks C.L., Scott A., Gispert Contamina I., Elani Y., Richardson R.J., Perriman A.W. (2023). Modular Bioorthogonal Lipid Nanoparticle Modification Platforms for Cardiac Homing. J. Am. Chem. Soc..

[36] Paulis L.E., Geelen T., Kuhlmann M.T., Coolen B.F., Schäfers M., Nicolay K., Strijkers G.J. (2012). Distribution of lipid-based nanoparticles to infarcted myocardium with potential application for MRI-monitored drug delivery. J. Control. Release.

[37] Shade C.W. (2016). Liposomes as Advanced Delivery Systems for Nutraceuticals. Integr. Med..

[38] Gök Yurttaş A., Gökduman K., Hekim N. (2022). Liposomes loaded with activatable disulfide bridged photosensitizer: Towards targeted and effective photodynamic therapy on breast cancer cells. Biointerface Res. Appl. Chem..

[39] Mukhamadiyarov R.A., Senokosova E.A., Krutitsky S.S., Voevoda D.V., Pyshnaya I.A., Ivanov V.V., Lewis M.J., Khaliulin I. (2018). Size-Dependent Ability of Liposomes to Accumulate in the Ischemic Myocardium and Protect the Heart. J. Cardiovasc. Pharmacol..

[40] Bowey K., Md J.-F., Tabrizian M. (2012). Liposome technology for cardiovascular disease treatment and diagnosis. Expert Opin. Drug Deliv..

[41] Wang S., Chen Y., Guo J., Huang Q. (2023). Liposomes for Tumor Targeted Therapy: A Review. Int. J. Mol. Sci..

[42] Mitragotri S., Patrick S. (2014). Organic nanoparticles for drug delivery and imaging. MRS Bull..

[43] Barenholz Y.C. (2012). Doxil^®^—The first FDA-approved nano-drug: Lessons learned. J. Control. Release.

[44] Geng Y.A.N., Dalhaimer P., Cai S., Tsai R., Tewari M., Minko T., Discher D.E. (2007). Shape effects of filaments versus spherical particles in flow and drug delivery. Nat. Nanotechnol..

[45] Nsairat H., Khater D., Sayed U., Odeh F., Al Bawab A., Alshaer W. (2022). Liposomes: Structure, composition, types, and clinical applications. Heliyon.

[46] Liu C., Zhang X., Yang H., Zhao M., Liu Y., Zhao R., Li Z., Sun M. (2024). PEG-modified nano liposomes co-deliver Apigenin and RAGE-siRNA to protect myocardial ischemia injury. Int. J. Pharm..

[47] Levchenko T.S., Hartner W.C., Torchilin V.P. (2012). Liposomes in diagnosis and treatment of cardiovascular disorders. Methodist DeBakey Cardiovasc. J..

[48] Wan J., Yang J., Lei W., Xiao Z., Zhou P., Zheng S., Zhu P. (2023). Anti-Oxidative, Anti-Apoptotic, and M2 Polarized DSPC Liposome Nanoparticles for Selective Treatment of Atherosclerosis. Int. J. Nanomed..

[49] Eloy J.O., Petrilli R., Trevizan L.N.F., Chorilli M. (2017). Immunoliposomes: A review on functionalization strategies and targets for drug delivery. Colloids Surf. B: Biointerfaces.

[50] Skourtis D., Stavroulaki D., Athanasiou V., Fragouli P.G., Iatrou H. (2020). Nanostructured polymeric, liposomal and other materials to control the drug delivery for cardiovascular diseases. Pharmaceutics.

[51] Chandarana M., Curtis A., Hoskins C. (2018). The use of nanotechnology in cardiovascular disease. Appl. Nanosci..

[52] Urban D., Pöss J., Böhm M., Laufs U. (2013). Targeting the Proprotein Convertase Subtilisin/Kexin Type 9 for the Treatment of Dyslipidemia and Atherosclerosis. J. Am. Coll. Cardiol..

[53] Niculescu A.-G., Bîrcă A.C., Grumezescu A.M. (2021). New Applications of Lipid and Polymer-Based Nanoparticles for Nucleic Acids Delivery. Pharmaceutics.

[54] Turnbull I.C., Eltoukhy A.A., Fish K.M., Hajjar R.J., Anderson D.G., Costa K.D. (2013). Myocardial Delivery of Lipidoid Nanoparticle mRNA Designed for Tailored Expression of Cardiogenic Factors. Circulation.

[55] Conway A., Mendel M., Kim K., McGovern K., Boyko A., Zhang L., Miller J.C., DeKelver R.C., Paschon D.E., Mui B.L. (2019). Non-viral delivery of zinc finger nuclease mRNA enables highly efficient in vivo genome editing of multiple therapeutic gene targets. Mol. Ther..

[56] Li Y., Zhou G., Bruno I.G., Cooke J.P. (2017). Telomerase mRNA reverses senescence in progeria cells. J. Am. Coll. Cardiol..

[57] Chanda P.K., Sukhovershin R., Cooke J.P. (2021). mRNA-enhanced cell therapy and cardiovascular regeneration. Cells.

[58] Kulkarni P., Rawtani D., Kumar M., Lahoti S.R. (2020). Cardiovascular drug delivery: A review on the recent advancements in nanocarrier based drug delivery with a brief emphasis on the novel use of magnetoliposomes and extracellular vesicles and ongoing clinical trial research. J. Drug Deliv. Sci. Technol..

[59] Al-Darraji A., Donahue R.R., Tripathi H., Peng H., Levitan B.M., Chelvarajan L., Haydar D., Gao E., Henson D., Gensel J.C. (2020). Liposomal delivery of azithromycin enhances its immunotherapeutic efficacy and reduces toxicity in myocardial infarction. Sci. Rep..

[60] Seidlitz A. (2019). Drug-Eluting Stents. In Vitro Drug Release Testing of Special Dosage Forms.

[61] Chen J., Song Y., Wang Q., Li Q., Tan H., Gao J., Zhang N., Weng X., Sun D., Yakufu W. (2022). Targeted neutrophil-mimetic liposomes promote cardiac repair by adsorbing proinflammatory cytokines and regulating the immune microenvironment. J. Nanobiotechnology.

[62] Brito L.A., Chandrasekhar S., Little S.R., Amiji M.M. (2010). In vitro and in vivo studies of local arterial gene delivery and transfection using lipopolyplexes-embedded stents. J. Biomed. Mater. Res. Part A.

[63] Allijn I.E., Czarny B.M.S., Wang X., Chong S.Y., Weiler M., da Silva A.E., Metselaar J.M., Lam C.S.P., Pastorin G., de Kleijn D.P.V. (2017). Liposome encapsulated berberine treatment attenuates cardiac dysfunction after myocardial infarction. J. Control. Release.

[64] Ko Y.T., Hartner W.C., Kale A., Torchilin V.P. (2009). Gene delivery into ischemic myocardium by double-targeted lipoplexes with anti-myosin antibody and TAT peptide. Gene Ther..

[65] Verma D.D., Levchenko T.S., Bernstein E.A., Torchilin V.P. (2005). ATP-loaded liposomes effectively protect mechanical functions of the myocardium from global ischemia in an isolated rat heart model. J. Control. Release Off. J. Control. Release Soc..

[66] Verma D., Levchenko T., Bernstein E., Mongayt D., Torchilin V. (2006). ATP-loaded immunoliposomes specific for cardiac myosin provide improved protection of the mechanical functions of myocardium from global ischemia in an isolated rat heart model. J. Drug Target..

[67] Harel-Adar T., Mordechai T.B., Amsalem Y., Feinberg M.S., Leor J., Cohen S. (2011). Modulation of cardiac macrophages by phosphatidylserine-presenting liposomes improves infarct repair. Proc. Natl. Acad. Sci. USA.

[68] Bertrand N., Bouvet C., Moreau P., Leroux J.-C. (2010). Transmembrane pH-Gradient Liposomes To Treat Cardiovascular Drug Intoxication. ACS Nano.

[69] Gyöngyösi M., Lukovic D., Zlabinger K., Spannbauer A., Gugerell A., Pavo N., Traxler D., Pils D., Maurer G., Jakab A. (2020). Liposomal doxorubicin attenuates cardiotoxicity via induction of interferon-related DNA damage resistance. Cardiovasc. Res..

[70] Ho D., Lynd T.O., Jun C., Shin J., Millican R.C., Estep B.K., Chen J., Zhang X., Brott B.C., Kim D.W. (2023). MiR-146a encapsulated liposomes reduce vascular inflammatory responses through decrease of ICAM-1 expression, macrophage activation, and foam cell formation. Nanoscale.

[71] Dorostkar H., Haghiralsadat B.F., Hemati M., Safari F., Hassanpour A., Naghib S.M., Roozbahani M.H., Mozafari M.R., Moradi A. (2023). Reduction of Doxorubicin-Induced Cardiotoxicity by Co-Administration of Smart Liposomal Doxorubicin and Free Quercetin: In Vitro and In Vivo Studies. Pharmaceutics.

[72] Onishchenko N.R., Moskovtsev A.A., Kobanenko M.K., Tretiakova D.S., Alekseeva A.S., Kolesov D.V., Mikryukova A.A., Boldyrev I.A., Kapkaeva M.R., Shcheglovitova O.N. (2023). Protein Corona Attenuates the Targeting of Antitumor Sialyl Lewis X-Decorated Liposomes to Vascular Endothelial Cells under Flow Conditions. Pharmaceutics.

[73] Alhaja M., Chen S., Chin A.C., Schulte B., Legasto C.S., Chin A., Legasto C. (2023). Cardiac Safety of Pegylated Liposomal Doxorubicin After Conventional Doxorubicin Exposure in Patients with Sarcoma and Breast Cancer. Cureus.

[74] Yusuf A., Almotairy A.R.Z., Henidi H., Alshehri O.Y., Aldughaim M.S. (2023). Nanoparticles as Drug Delivery Systems: A Review of the Implication of Nanoparticles’ Physicochemical Properties on Responses in Biological Systems. Polymers.

[75] Woodside D.G., Tanifum E.A., Ghaghada K.B., Biediger R.J., Caivano A.R., Starosolski Z.A., Khounlo S., Bhayana S., Abbasi S., Craft J.W. (2018). Magnetic Resonance Imaging of Atherosclerotic Plaque at Clinically Relevant Field Strengths (1T) by Targeting the Integrin α4β1. Sci. Rep..

[76] Ayyagari A.L., Zhang X., Ghaghada K.B., Annapragada A., Hu X., Bellamkonda R.V. (2006). Long-circulating liposomal contrast agents for magnetic resonance imaging. Magn. Reson. Med..

[77] Howles G.P., Ghaghada K.B., Qi Y., Mukundan S., Johnson G.A. (2009). High-resolution magnetic resonance angiography in the mouse using a nanoparticle blood-pool contrast agent. Magn. Reson. Med..

[78] Maiseyeu A., Mihai G., Kampfrath T., Simonetti O.P., Sen C.K., Roy S., Rajagopalan S., Parthasarathy S. (2009). Gadolinium-containing phosphatidylserine liposomes for molecular imaging of atherosclerosis. J. Lipid Res..

[79] Wrobeln A., Schlüter K.D., Linders J., Zähres M., Mayer C., Kirsch M., Ferenz K.B. (2017). Functionality of albumin-derived perfluorocarbon-based artificial oxygen carriers in the Langendorff-heart. Artif. Cells Nanomed. Biotechnol..

[80] Cheng M., Liu Q., Liu W., Yuan F., Feng J., Jin Y., Tu L. (2021). Engineering micelles for the treatment and diagnosis of atherosclerosis. J. Drug Deliv. Sci. Technol..

[81] Yang F., Xue J., Wang G., Diao Q. (2022). Nanoparticle-based drug delivery systems for the treatment of cardiovascular diseases. Front. Pharmacol..

[82] Peters D., Kastantin M., Kotamraju V.R., Karmali P.P., Gujraty K., Tirrell M., Ruoslahti E. (2009). Targeting atherosclerosis by using modular, multifunctional micelles. Proc. Natl. Acad. Sci. USA.

[83] Sabir F., Barani M., Mukhtar M., Rahdar A., Cucchiarini M., Zafar M.N., Behl T., Bungau S. (2021). Nanodiagnosis and Nanotreatment of Cardiovascular Diseases: An Overview. Chemosensors.

[84] Wu B., Huang W.-Q., Nie X., Zhang Z., Chen G., Wang H.-L., Wang F., Ding S.-G., Hao Z.-Y., You Y.-Z. (2021). The effect of topology of PEG chain on the stability of micelles in brine and serum. Colloid Interface Sci. Commun..

[85] Beilvert A., Cormode D.P., Chaubet F., Briley-Saebo K.C., Mani V., Mulder W.J., Vucic E., Toussaint J.F., Letourneur D., Fayad Z.A. (2009). Tyrosine polyethylene glycol (PEG)-micelle magnetic resonance contrast agent for the detection of lipid rich areas in atherosclerotic plaque. Magn. Reson. Med..

[86] Cormode D.P., Naha P.C., Fayad Z.A. (2014). Nanoparticle contrast agents for computed tomography: A focus on micelles. Contrast Media Mol. Imaging.

[87] Karademir F., Ayhan F. (2021). Antimicrobial Surface Functionality of PEG Coated and AgNPs Immobilized Extracorporeal Biomaterials. Biointerface Res. Appl. Chem.

[88] Szebeni J., Alving C., Rosivall L., Bünger R., Baranyi L., Bedocs P., Tóth M., Barenholz Y. (2007). Animal Models of Complement-Mediated Hypersensitivity Reactions to Liposomes and Other Lipid-Based Nanoparticles. J. Liposome Res..

[89] Szebeni J. (2005). Complement activation-related pseudoallergy: A new class of drug-induced acute immune toxicity. Toxicology.

[90] Nakashiro S., Matoba T., Umezu R., Koga J., Tokutome M., Katsuki S., Nakano K., Sunagawa K., Egashira K. (2016). Pioglitazone-Incorporated Nanoparticles Prevent Plaque Destabilization and Rupture by Regulating Monocyte/Macrophage Differentiation in ApoE^−/−^ Mice. Arter. Thromb. Vasc. Biol..

[91] Chin D.D., Poon C., Wang J., Joo J., Ong V., Jiang Z., Cheng K., Plotkin A., Magee G.A., Chung E.J. (2021). miR-145 micelles mitigate atherosclerosis by modulating vascular smooth muscle cell phenotype. Biomaterials.

[92] Wennink J.W.H., Liu Y., Mäkinen P.I., Setaro F., de la Escosura A., Bourajjaj M., Lappalainen J.P., Holappa L.P., van den Dikkenberg J.B., Al Fartousi M. (2017). Macrophage selective photodynamic therapy by meta-tetra(hydroxyphenyl)chlorin loaded polymeric micelles: A possible treatment for cardiovascular diseases. Eur. J. Pharm. Sci. Off. J. Eur. Fed. Pharm. Sci..

[93] Yoo S.P., Pineda F., Barrett J.C., Poon C., Tirrell M., Chung E.J. (2016). Gadolinium-Functionalized Peptide Amphiphile Micelles for Multimodal Imaging of Atherosclerotic Lesions. ACS Omega.

[94] Kirana C., Rogers P.F., Bennett L.E., Abeywardena M.Y., Patten G.S. (2005). Naturally derived micelles for rapid in vitro screening of potential cholesterol-lowering bioactives. J. Agric. Food Chem..

[95] Yu S.S., Lau C.M., Barham W.J., Onishko H.M., Nelson C.E., Li H., Smith C.A., Yull F.E., Duvall C.L., Giorgio T.D. (2013). Macrophage-Specific RNA Interference Targeting via “Click”, Mannosylated Polymeric Micelles. Mol. Pharm..

[96] Hyatt D., Chung E.J., Tirrell M., Alenghat F.J. (2014). Monocyte and macrophage-directed peptide amphiphile micelles modulate cytoskeletal organization and target atherosclerosis. Circulation.

[97] Chin D.D., Poon C., Trac N., Wang J., Cook J., Joo J., Jiang Z., Sta Maria N.S., Jacobs R.E., Chung E.J. (2020). Collagenase-cleavable peptide Amphiphile micelles as a novel Theranostic strategy in atherosclerosis. Adv. Ther..

[98] Kuo C.H., Leon L., Chung E.J., Huang R.T., Sontag T.J., Reardon C.A., Getz G.S., Tirrell M., Fang Y. (2014). Inhibition of atherosclerosis-promoting microRNAs via targeted polyelectrolyte complex micelles. J. Mater. Chem. B.

[99] Nguyen L.T.H., Muktabar A., Tang J., Dravid V.P., Thaxton C.S., Venkatraman S., Ng K.W. (2017). Engineered nanoparticles for the detection, treatment and prevention of atherosclerosis: How close are we?. Drug Discov. Today.

[100] Gupta M.K., Martin J.R., Werfel T.A., Shen T., Page J.M., Duvall C.L. (2014). Cell Protective, ABC Triblock Polymer-Based Thermoresponsive Hydrogels with ROS-Triggered Degradation and Drug Release. J. Am. Chem. Soc..

[101] Allen S.D., Liu Y.-G., Kim T., Bobbala S., Yi S., Zhang X., Choi J., Scott E.A. (2019). Celastrol-loaded PEG-b-PPS nanocarriers as an anti-inflammatory treatment for atherosclerosis. Biomater. Sci..

[102] Chin D.D., Wang J., Mel de Fontenay M., Plotkin A., Magee G.A., Chung E.J. (2019). Hydroxyapatite-binding micelles for the detection of vascular calcification in atherosclerosis. J. Mater. Chem. B.

[103] Chen J., Zhang X., Millican R., Sherwood J., Martin S., Jo H., Yoon Y.-s., Brott B.C., Jun H.-W. (2021). Recent advances in nanomaterials for therapy and diagnosis for atherosclerosis. Adv. Drug Deliv. Rev..

[104] Soumya R.S., Raghu K.G. (2023). Recent advances on nanoparticle-based therapies for cardiovascular diseases. J. Cardiol..

[105] Shen Y., Yu X., Cui J., Yu F., Liu M., Chen Y., Wu J., Sun B., Mo X. (2022). Development of Biodegradable Polymeric Stents for the Treatment of Cardiovascular Diseases. Biomolecules.

[106] Hsissou R., Hilali M., Dagdag O., Adder F., Elbachiri A., Rafik M. (2021). Rheological behavior models of polymers. Biointerface Res. Appl. Chem..

[107] Al Meslmani B.M., Mahmoud G.F., Bakowsky U. (2017). Development of expanded polytetrafluoroethylene cardiovascular graft platform based on immobilization of poly lactic-co-glycolic acid nanoparticles using a wet chemical modification technique. Int. J. Pharm..

[108] Ahadian S., Davenport Huyer L., Estili M., Yee B., Smith N., Xu Z., Sun Y., Radisic M. (2017). Moldable elastomeric polyester-carbon nanotube scaffolds for cardiac tissue engineering. Acta Biomater..

[109] Chang M.Y., Yang Y.J., Chang C.H., Tang A.C., Liao W.Y., Cheng F.Y., Yeh C.S., Lai J.J., Stayton P.S., Hsieh P.C. (2013). Functionalized nanoparticles provide early cardioprotection after acute myocardial infarction. J. Control. Release Off. J. Control. Release Soc..

[110] Dvir T., Bauer M., Schroeder A., Tsui J.H., Anderson D.G., Langer R., Liao R., Kohane D.S. (2011). Nanoparticles Targeting the Infarcted Heart. Nano Lett..

[111] Ren Y., Wang X., Liang H., He W., Zhao X. (2021). Mechanism of miR-30b-5p-Loaded PEG-PLGA Nanoparticles for Targeted Treatment of Heart Failure. Front. Pharmacol..

[112] Ma Q., Yang J., Huang X., Guo W., Li S., Zhou H., Li J., Cao F., Chen Y. (2018). Poly(Lactide-Co-Glycolide)-Monomethoxy-Poly-(Polyethylene Glycol) Nanoparticles Loaded with Melatonin Protect Adipose-Derived Stem Cells Transplanted in Infarcted Heart Tissue. Stem Cells.

[113] Kesavan S., Meena K.S., Dhakshinamoorthy R. (2022). Bioactive polysaccharides based graphene oxide nanoparticle as a promising carrier for anticancer drug delivery. Biointerface Res. Appl. Chem.

[114] Shi W., Fuad A.R.M., Li Y., Wang Y., Huang J., Du R., Wang G., Wang Y., Yin T. (2023). Biodegradable polymeric nanoparticles increase risk of cardiovascular diseases by inducing endothelium dysfunction and inflammation. J. Nanobiotechnology.

[115] Sun K., Yuan R., He J., Zhuo Y., Yang M., Hao E., Hou X., Yao C., Yang S., Gao H. (2023). Sugarcane leaf polysaccharide exerts a therapeutic effect on cardiovascular diseases through necroptosis. Heliyon.

[116] Dormont F., Varna M., Couvreur P. (2018). Nanoplumbers: Biomaterials to fight cardiovascular diseases. Mater. Today.

[117] Luong-Van E., Grøndahl L., Chua K.N., Leong K.W., Nurcombe V., Cool S.M. (2006). Controlled release of heparin from poly(ε-caprolactone) electrospun fibers. Biomaterials.

[118] Karam M., Fahs D., Maatouk B., Safi B., Jaffa A.A., Mhanna R. (2022). Polymeric nanoparticles in the diagnosis and treatment of myocardial infarction: Challenges and future prospects. Mater. Today. Bio.

[119] Wang Y.-J., Larsson M., Huang W.-T., Chiou S.-H., Nicholls S.J., Chao J.-I., Liu D.-M. (2016). The use of polymer-based nanoparticles and nanostructured materials in treatment and diagnosis of cardiovascular diseases: Recent advances and emerging designs. Prog. Polym. Sci..

[120] Leal B.B.J., Wakabayashi N., Oyama K., Kamiya H., Braghirolli D.I., Pranke P. (2021). Vascular Tissue Engineering: Polymers and Methodologies for Small Caliber Vascular Grafts. Front. Cardiovasc. Med..

[121] Jin G., Gao Z., Liu Y., Zhao J., Ou H., Xu F., Ding D. (2021). Polymeric Nitric Oxide Delivery Nanoplatforms for Treating Cancer, Cardiovascular Diseases, and Infection. Adv. Healthc. Mater..

[122] Li X., Hong G., Zhao G., Pei H., Qu J., Chun C., Huang Z., Lu Z. (2022). Red Blood Cell Membrane-Camouflaged PLGA Nanoparticles Loaded With Basic Fibroblast Growth Factor for Attenuating Sepsis-Induced Cardiac Injury. Front. Pharmacol..

[123] Oh K.S., Song J.Y., Yoon S.J., Park Y., Kim D., Yuk S.H. (2010). Temperature-induced gel formation of core/shell nanoparticles for the regeneration of ischemic heart. J. Control. Release Off. J. Control. Release Soc..

[124] Nenna A., Nappi F., Larobina D., Verghi E., Chello M., Ambrosio L. (2021). Polymers and Nanoparticles for Statin Delivery: Current Use and Future Perspectives in Cardiovascular Disease. Polymers.

[125] Pechanova O., Dayar E., Cebova M. (2020). Therapeutic potential of polyphenols-loaded polymeric nanoparticles in cardiovascular system. Molecules.

[126] Kim S.M., Patel M., Patel R. (2021). PLGA Core-Shell Nano/Microparticle Delivery System for Biomedical Application. Polymers.

[127] Banik B.L., Fattahi P., Brown J.L. (2016). Polymeric nanoparticles: The future of nanomedicine. Wiley Interdiscip. Rev. Nanomed. Nanobiotechnology.

[128] Jarai B.M., Kolewe E.L., Stillman Z.S., Raman N., Fromen C.A. (2020). Polymeric nanoparticles. Nanoparticles for Biomedical Applications.

[129] Wang W., Chen J., Li M., Jia H., Han X., Zhang J., Zou Y., Tan B., Liang W., Shang Y. (2019). Rebuilding Postinfarcted Cardiac Functions by Injecting TIIA@PDA Nanoparticle-Cross-linked ROS-Sensitive Hydrogels. ACS Appl. Mater. Interfaces.

[130] Shapoval O., Brandmeier J.C., Nahorniak M., Oleksa V., Makhneva E., Gorris H.H., Farka Z., Horák D. (2022). PMVEMA-coated upconverting nanoparticles for upconversion-linked immunoassay of cardiac troponin. Talanta.

[131] He Y., Ye G., Song C., Li C., Xiong W., Yu L., Qiu X., Wang L. (2018). Mussel-inspired conductive nanofibrous membranes repair myocardial infarction by enhancing cardiac function and revascularization. Theranostics.

[132] Wang L., Jiang J., Hua W., Darabi A., Song X., Song C., Zhong W., Xing M.M.Q., Qiu X. (2016). Mussel-Inspired Conductive Cryogel as Cardiac Tissue Patch to Repair Myocardial Infarction by Migration of Conductive Nanoparticles. Adv. Funct. Mater..

[133] Zenych A., Jacqmarcq C., Aid R., Fournier L., Forero Ramirez L.M., Chaubet F., Bonnard T., Vivien D., Letourneur D., Chauvierre C. (2021). Fucoidan-functionalized polysaccharide submicroparticles loaded with alteplase for efficient targeted thrombolytic therapy. Biomaterials.

[134] Tetali S.S.V., Fricker A.T.R., van Domburg Y.A., Roy I. (2023). Intelligent biomaterials for cardiovascular applications. Curr. Opin. Biomed. Eng..

[135] Chung T.W., Wang S.S., Tsai W.J. (2008). Accelerating thrombolysis with chitosan-coated plasminogen activators encapsulated in poly-(lactide-co-glycolide) (PLGA) nanoparticles. Biomaterials.

[136] Yu M., Zhu Y., Lin C.-J., Wang S., Xing J., Jang C., Huang J., Huang J., Jin J., Yu L. (2019). Effects of air pollution control measures on air quality improvement in Guangzhou, China. J. Environ. Manag..

[137] Beldman T.J., Senders M.L., Alaarg A., Pérez-Medina C., Tang J., Zhao Y., Fay F., Deichmöller J., Born B., Desclos E. (2017). Hyaluronan Nanoparticles Selectively Target Plaque-Associated Macrophages and Improve Plaque Stability in Atherosclerosis. ACS Nano.

[138] Zhang S.-F., Gao C., Lü S., He J., Liu M., Wu C., Liu Y., Zhang X., Liu Z. (2017). Synthesis of PEGylated polyglutamic acid peptide dendrimer and its application in dissolving thrombus. Colloids Surf. B: Biointerfaces.

[139] Schulz R., Kelm M., Heusch G. (2004). Nitric oxide in myocardial ischemia/reperfusion injury. Cardiovasc. Res..

[140] Bolli R., Becker L., Gross G., Mentzer Jr R., Balshaw D., Lathrop D.A. (2004). Myocardial protection at a crossroads: The need for translation into clinical therapy. Circ. Res..

[141] Hogg N. (2000). Biological chemistry and clinical potential of S-nitrosothiols. Free Radic. Biol. Med..

[142] Stasko N.A., Fischer T.H., Schoenfisch M.H. (2008). S-nitrosothiol-modified dendrimers as nitric oxide delivery vehicles. Biomacromolecules.

[143] Kulhari H., Pooja D., Prajapati S., Chauhan A.S. (2011). Performance evaluation of PAMAM dendrimer based simvastatin formulations. Int. J. Pharm..

[144] Devarakonda B., Hill R.A., de Villiers M.M. (2004). The effect of PAMAM dendrimer generation size and surface functional group on the aqueous solubility of nifedipine. Int. J. Pharm..

[145] Singh M.K., Pooja D., Kulhari H., Jain S.K., Sistla R., Chauhan A.S. (2017). Poly (amidoamine) dendrimer-mediated hybrid formulation for combination therapy of ramipril and hydrochlorothiazide. Eur. J. Pharm. Sci. Off. J. Eur. Fed. Pharm. Sci..

[146] Fu Y., Nitecki D.E., Maltby D., Simon G.H., Berejnoi K., Raatschen H.-J., Yeh B.M., Shames D.M., Brasch R.C. (2006). Dendritic iodinated contrast agents with PEG-cores for CT imaging: Synthesis and preliminary characterization. Bioconjugate Chem..

[147] Ardestani M.S., Bitarafan-Rajabi A., Mohammadzadeh P., Mortazavi-Derazkola S., Sabzevari O., Azar A.D., Kazemi S., Hosseini S.R., Ghoreishi S.M. (2020). Synthesis and characterization of novel 99mTc-DGC nano-complexes for improvement of heart diagnostic. Bioorganic Chem..

[148] Ferrario C.M., Strawn W.B. (2006). Role of the renin-angiotensin-aldosterone system and proinflammatory mediators in cardiovascular disease. Am. J. Cardiol..

[149] Liu J., Gu C., Cabigas E.B., Pendergrass K.D., Brown M.E., Luo Y., Davis M.E. (2013). Functionalized dendrimer-based delivery of angiotensin type 1 receptor siRNA for preserving cardiac function following infarction. Biomaterials.

[150] Xue X., Shi X., Dong H., You S., Cao H., Wang K., Wen Y., Shi D., He B., Li Y. (2018). Delivery of microRNA-1 inhibitor by dendrimer-based nanovector: An early targeting therapy for myocardial infarction in mice. Nanomed. Nanotechnol. Biol. Med..

[151] Wang Z. (2010). MicroRNA: A matter of life or death. World J. Biol. Chem..

[152] Gothwal A., Kesharwani P., Gupta U., Khan I., Iqbal Mohd Amin M.C., Banerjee S., Iyer A.K. (2015). Dendrimers as an Effective Nanocarrier in Cardiovascular Disease. Curr. Pharm. Des..

